# SARS-CoV-2 Infection Influences Wnt/β-Catenin Pathway Components in Astrocytes

**DOI:** 10.3390/pathogens14100994

**Published:** 2025-10-02

**Authors:** KaReisha F. Robinson, Avantika I. Ahiya, Justin M. Richner, Sarah E. Lutz

**Affiliations:** 1 Department of Anatomy and Cell Biology, University of Illinois Chicago, Chicago, IL 60612, USA; krobi01s@uic.edu (K.F.R.); aa208@uic.edu (A.I.A.); 2 Department of Microbiology and Immunology, University of Illinois Chicago, Chicago, IL 60612, USA

**Keywords:** Wnt/β-catenin, astrocytes, SARS-CoV-2

## Abstract

The mechanisms by which SARS-CoV-2 infection lead to neuroinflammation and cognitive impairment in COVID-19 and Long COVID are unclear. Cerebrovascular Wnt/β-catenin pathway activity is suppressed in association with neuroinflammation and cognitive impairment in a mouse model of COVID-19. In this study, we asked whether SARS-CoV-2 (NY Iota strain) infection of astrocytes would result in cell-autonomous changes in Wnt/β-catenin pathway components. We report that induced pluripotent stem cell (hiPSC)-derived human astrocytes (iAs) are susceptible to sustained infection with SARS-CoV-2 in vitro. Real-time PCR revealed that SARS-CoV-2 infection of iAs decreased transcripts for Wnt3a, Wnt10b, and the downstream pathway effectors β-catenin and TCF3. Wnt7b was increased, as was the proinflammatory chemokine CXCL10. No changes were noted in Wnt3, Wnt7a, TCF1, TCF4, or LEF1. These data indicate that SARS-CoV-2 infection differentially influences Wnt/β-catenin pathway components in astrocytes. These data could have implications for the mechanistic basis of COVID-19 and Long COVID.

## 1. Introduction

SARS-CoV-2 cases have totaled over 700 million worldwide with over 5 million deaths. Throughout the pandemic, many patients infected with SARS-CoV-2 experienced neurological symptoms such as loss of sense of smell and taste, headaches, hypoxia, neuroinflammation, cerebrovascular pathology, and encephalitis [1,2,3]. At least 10% of SARS-CoV-2 patients develop Long COVID, often including neurocognitive impairments like brain fog and memory loss [4,5,6]. The causes of Long COVID are incompletely understood, but could involve infection of CNS cells. However, the extent to which brain cells are directly infected with SARS-CoV-2 is controversial. Indeed, reports have [2,3,6,7,8,9,10,11,12,13] or have not [14,15] found evidence of SARS-CoV-2 infection in the brain. Therefore, the extent to which SARS-CoV-2 infection/exposure of neural cells contributes to neurologic disease is an area of ongoing investigation. Some data supports that astrocytes can be infected with SARS-CoV-2 [1,16,17] and SARS-CoV-2 viral products induce potent cellular neuroinflammatory responses [1,8,11,18,19,20]. Indeed, astrocytes robustly express multiple SARS-CoV-2 receptors [21]. Astrocyte infection or exposure to viral products could contribute to COVID-19/Long COVID neuropathogenesis by altering key homeostatic or inflammatory pathways in the brain [1,2,11,22,23,24].

COVID-19/Long COVID neurologic deficits are strongly associated with blood-brain barrier (BBB) permeability and dysregulation of neurogenesis [25,26,27]. The Wnt/β-catenin pathway is important for BBB integrity [28,29,30,31,32,33,34,35,36,37]. Interestingly, several studies support that the Wnt/β-catenin pathway may be disrupted in the brain in COVID-19 patients and models, as evidenced by elevation of Wnt/β-catenin pathway inhibitors and reduction in expression of Wnt/β-catenin transcriptional targets [22,38,39,40,41]. Wnt/β-catenin pathway activation increases brain endothelial cell barrier properties and influences cell proliferation [42,43,44,45,46]. Therefore, alterations in Wnt/β-catenin signaling could influence these processes in COVID-19. Indeed, we previously reported that cerebrovascular-targeted delivery of an engineered Wnt7a ligand improved BBB integrity, neuroinflammation, and cognitive function in mouse-adapted SARS-CoV-2 (MA10), a mouse model of COVID-19 [40].

Canonical Wnt/β-catenin signaling is initiated by Wnt binding to a receptor complex minimally consisting of a Frizzled seven transmembrane protein, and low-density lipoprotein-related protein 5/6 (LRP 5/6) [47,48]. This interaction causes the β-catenin destruction complex (Axin, Disheveled (DVL), casein kinase-1α (CK-1α), and glycogen synthase kinase-3β (GSK-3β)) to dissociate from LRP 5/6 due to its phosphorylation by CK-1α and GSK-3β [47,48]. Then β-catenin becomes stabilized due to hypophosphorylation and translocates into the nucleus. β-catenin binds to the TCF/LEF complex, recruits transcription factors such as Pygo, Bcl9, and CBP/p300, and regulates gene expression [47,48]. Wnt/β-catenin signaling is known to be disrupted in other neurological diseases including Human Immunodeficiency Virus (HIV)-Associated Neurocognitive Disorders (HAND) [47,49,50,51], Zika [52], Multiple Sclerosis (MS) [53,54], Alzheimer’s disease (AD) [55,56,57], Parkinson’s disease (PD) [58,59], and Huntington’s disease (HD) [60].

Astrocytes participate in the Wnt/β-catenin pathway by secreting and by receiving Wnt ligands. For example, astrocyte secretion of Wnt ligands critically regulate BBB properties of brain endothelial cells [28,61,62] and neurogenesis [63]. Astrocytes also respond to Wnt ligands through activation of the Wnt/β-catenin pathway, with notable effects including suppression of proinflammatory cytokines such as IFNγ [64,65,66], granulocyte–macrophage colony-stimulating factor (GM-CSF) [64,67], and TNFα [68]. Previous studies have shown that canonical Wnt signaling components such as β-catenin and TCFs-1, -3, and -4 are expressed in astrocytes [47,69]. However, these are downregulated in astrocytes infected with HIV or treated with the virally induced cytokine IFNγ [47,70,71]. Importantly, downregulation of canonical signaling components within astrocytes de-represses astrocyte production of IL-6 [68] (a pleiotropic cytokine) [68,72,73,74] and IL-8 [75] (a chemoattractant for leukocyte trafficking into the CNS) [75,76,77]. Previous studies have shown that downregulation of β-catenin leads to astrocytic senescence, and eventually, neuronal injury [78,79].

Here, we explored how SARS-CoV-2 infection modulates the Wnt/β-catenin pathway in astrocytes and their subsequent inflammatory reaction in vitro. We tested the extent to which astrocyte infection with SARS-CoV-2 would alter Wnt/β-catenin pathway activity, production of Wnt ligands, and production of the chemokine CXCL10. We used human inducible pluripotent stem cell (hiPSC)-derived astrocytes (iAs) because they replicate quickly, are transcriptomically consistent with astrocytes of other studies, easily assessable, cost-effective, and robustly express the Wnt/β-catenin pathway [48,50,53,60,65,80,81]. We found that some components of the Wnt/β-catenin pathway (β-catenin, TCF3, Wnt3a, and Wnt10b) are downregulated in response to SARS-CoV-2 infection. Yet, Wnt7b was induced in response to SARS-CoV-2 infection. Furthermore, infection upregulated CXCL10. Changes in Wnt/β-catenin signaling in SARS-CoV-2-exposed astrocytes could shed light on potential neuropathogenic mechanisms of COVID-19/Long COVID.

## 2. Materials and Methods

### 2.1. Cell Culture

**Figure 1:**
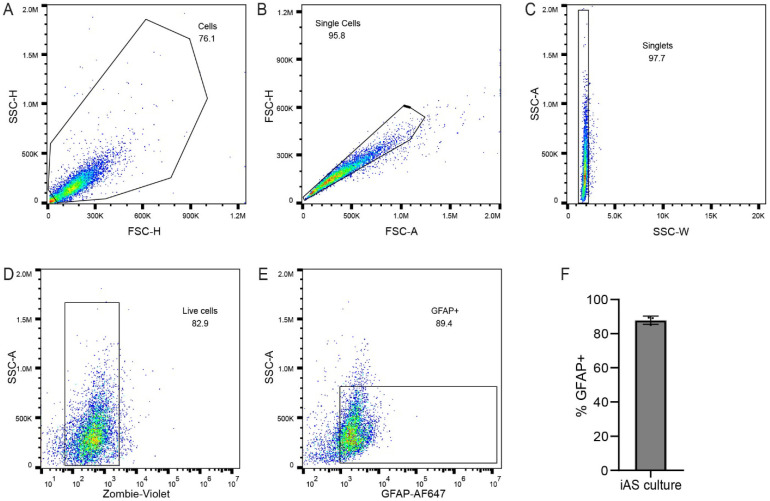
iAs cultures contain astrocytes. (**A**–**F**) Flow cytometry was used to probe iAs cultures to confirm astrocyte identity. Cell suspensions were gated by forward and side scatter for single cells (**A**–**C**). Vital dye exclusion was used to identify live cells (**D**). Live singlets were gated for positivity for the astrocyte marker GFAP (**E**). An average of 88% of live cells were positive for GFAP (**F**; range, 85.1–89.1%). The statistical analysis of (**F**) is detailed in Table S1.

Human inducible pluripotent stem cell (hiPSC)-derived astrocytes (iAs) were a generous gift from Dr. Lena Al-Harthi (Rush University Medical Center, Department of Microbial Pathogens and Immunity, Chicago, IL, USA). Differentiation and phenotypic characterization of iAs from hiPSC has been previously described by Dr. Al-Harthi’s group [81], and is assessed here (Figure 1). As before [81], the iAs were maintained and cultured in astrocyte medium (AM, Cat. #1801, ScienCell, Carlsbad, CA, USA) consisting of basal medium supplemented with 2% fetal bovine serum (FBS, Cat. #0010, ScienCell), 1% astrocyte growth medium supplement (AGS, Cat. #1801, ScienCell), and 1% penicillin/streptomycin (P/S, Cat. #0503, ScienCell) solution. Days 52–86 were used in these experiments. Cells were maintained in a 5% CO_2_ humidified atmosphere at 37 °C.

### 2.2. Virus and Viral Infections

The following reagent was obtained through BEI Resources, NIAID, NIH: SARS-Related Coronavirus 2, Isolate hCoV-19/USA/NY-NP-DOH1/2021 (Lineage B.1.526), NR-55359, contributed by Dr. David D. Ho, Columbia University. Virus was propagated for one passage on VeroE6 cells. iAs were ~90% confluent at the time of infection and used 24–96 h post infection for further downstream applications. Infection of virus was at 3 multiplicity of infection (MOI)/12-well format in infection media consisting of OptiMem (Cat. #31985070, ThermoFisher Scientific, Waltham, MA, USA) 1% P/S (Cat. #A5873601, ThermoFisher Scientific), and 2% FBS (Cat. # A5209401, ThermoFisher Scientific). Infection volume was at least 200 µL/well in a 12-well plate. When infecting, the iAs were maintained in a 5% CO_2_ humidified atmosphere at 37 °C, rocking every 15 min for 1 h. Afterward, virus was removed, the iAs were washed three times with warmed Phosphate Buffered Saline (PBS, Cat. #AM9625, ThermoFisher Scientific), fresh media was added, and iAs incubated in a 5% CO_2_ humidified atmosphere at 37 °C overnight. All infections were performed under Biosafety Level 3 (BSL3) containment.

### 2.3. Flow Cytometry

iAs treated with mock or SARS-CoV2 for 24 h were trypsinized for 5 mins and washed with iAs medium containing FBS. Cells were stained with Zombie-Violet (1:200, Biolegend Cat. #423113, San Diego, CA, USA) for 30 minutes and washed with 0.5% PBS + BSA. Following Fix/Perm, cells were stained with anti-GFAP-AF647 (1:50, Abcam, Cat.#AB279290, Cambridge, UK) and anti-β-catenin-PE (1:50, Biolegend Cat. #862603, Cambridge, UK) for 30 min, washed, and resuspended in 0.5% PBS + BSA and analyzed on CytoFlex S at UIC’s Flow Cytometry Core.

### 2.4. Quantitative Real-Time PCR

RNA was isolated using Tri Reagent (Sigma, St. Louis, MO, USA) as per manufacturer’s instructions. cDNA was synthesized using High-Capacity cDNA Reverse Transcription Kit (Cat. #4368814, ThermoFisher Scientific). Real-time PCR was performed using PowerUp Sybr Green Master Mix (Cat. #A25472, ThermoFisher Scientific) in a ViiA7 Real-Time PCR System using a 7500 software V2.0.6 at UIC’s Genome core. Reaction conditions were 50 °C for 2 min and 20 s, then 95 °C for 2 min; followed by 40 cycles of denaturation at 95 °C for 15 s, annealing and extension at 60 °C for 30 s, and a melt curve extension stage at 95 °C for 15 s, 60 °C for 1 min, and 95 °C for 15 s. Melting curve analysis was performed to ensure the amplification of a single product. Primers used were β-catenin–F-5′-CGTGCACATCAGGATACCCA-3′ and β-catenin–R-5′-GGCTCCGGTACAACCTTCAA-3′; Wnt3-F-5′-ACCTGCAAGTAGGGCACCAG-3′ and Wnt3-R-5′-GCGAGTTGGGTCTGGGTCAT-3′; Wnt3a-F-5′-TGGCCCCACTCGGATACTTC-3′ and Wnt3a-R-5′-GCGAGCCCAGGGAGGAATAC-3′; Wnt5a-F-5′-CTTCCGCAAGGTGGGTGAT-3′ and Wnt5a-R-5′- GAAGCGGCTGTTGACCTGTA-3′; Wnt7a-F-5′-GGTGCGAGCATCATCTGTAA-3′ and Wnt7a-R-5′-CATTTGGGAGCCTTCTCCTATG-3′; Wnt7b-F-5′-ATGTGTTCTTGAGCAGCCGA-3′ and Wnt7b-R-5′-CAGGCATGGTTAGAGGCACA-3′; Wnt10b-F-5′-GAATGCGGATCCACAACAAC-3′ and Wnt10b-R-5′-TGTGCCATGACACTTGCATTTCCG-3′; TCF1-F-5′-AGCGCTGCCATCAACCAGAT-3′ and TCF1-R-5′-CCTCCTGTGGTGGATTCTTGGT-3′; TCF3-F-5′-ATCCTGCACCAGGCTGTCTC-3′ and TCF3-R-5′-CCATCTGGGGGTCTCCAACC-3′; TCF4-F-5′-GCAGGGACCTTGGGTCACAT-3′ and TCF4-R-5′-AAGGAGACTCTGCTGGTGGC-3′; LEF1-F-5′-CCAGGCTGGTCTGCAAGAGA-3′ and LEF1-R-5′-GCAGCTGTCATTCTTGGACCT-3′; IL-6-F-5′-CTGCCTTCCCTGCCCCAGTA-3′ and IL-6-R-5′-TTCTGCCAGTGCCTCTTTGC-3′; IL-8-F-5′-CCACACTGCGCCAACACAG-3′ and IL-8-R-5′-TTCTCAGCCCTCTTCAAAAACTTC-3′; CCL2-F-5′-ACCTTCATTCCCCAAGGGCTC-3′ and CCL2-R-5′-GGACACTTGCTGCTGGTGAT-3′; CXCL10-F-5′-GGTGAGAAGAGATGTCTGAATCC and CXCL10-R-5′-GTCCATCCTTGGAAGCACTGCA-3′; GAPDH-F-5′-TCCTGTTCGACAGTCAGCCG and GAPDH-R-5′-CCCCATGGTGTCTGAGCGAT-3′. Fold change in mRNA expression was calculated by relative quantification using the comparative CT method with GAPDH as the endogenous control.

To measure viral infectivity, real-time PCR was performed using TaqMan RNA-to-CT 1-Step Kit (Cat. #4392938, ThermoFisher Scientific) in a ViiA7 Real-Time PCR System using a 7500 software V2.0.6 at UIC’s Genome core. Reaction conditions were reverse transcription at 48 °C for 15 min, then enzyme activation at 95 °C for 10 min; followed by 40 cycles of denaturation at 95 °C for 15 s and an annealing and extension step at 60 °C for 1 min. The CDC primer/probe kit (IDT; Cat. #10006713, Newark, NJ, USA) was used against the N1 gene. Genomes per milliliter were interpolated using CT values and genomic standard (BEI Resources; Cat #. NR-52358, NIAID, Bethesda, MD, USA) and were run in triplicate.

### 2.5. Viral Quantification via Focus-Forming Assay

Vero cells were plated at 2 × 10^4^ cells per well in 100 μL in 96-well TC plates. In the BSL3 facility, samples were diluted in a round bottom 96-well plate by adding 50 μL of sample into 100 μL of Dulbecco’s Modified Eagle Medium (DMEM, Cat. # 30-2002, ATCC, Manassas, VA, USA) + 2% FBS media, making serial 3-fold dilutions. Media was removed and 100 μL/well of NY Iota SARS-CoV-2 virus strain was added to the Vero cells in duplicate, rocking the plate from side-to-side. The Vero cells with virus incubated in 37 °C for 1 h. A total of ~125 μL/well of overlay (1% methylcellulose in 1× MEM + 2% FBS) was added to the Vero cells with virus and incubated overnight at 37 °C for 24 h. Vero cells with virus were fixed in 4% paraformaldehyde (PFA, Cat#: J19943.K2, ThermoFisher Scientific) diluted in PBS at 100 μL/well. Once removed from the BSL3 facility, the cells were washed three times with PBS. A total of 1:150,000 (1 ug/mL) SARS guinea pig primary antibody (BEI Resources; Cat #. NR10361, NIAID, Bethesda, MD, USA) diluted in PermWash (0.1% saponin (Cat #. J63209.AK, ThermoFisher Scientific) and 0.1% bovine serum albumin (BSA, Cat #. B14, ThermoFisher Scientific) in PBS was added at 100 μL/well and incubated at 4 °C rocking overnight. The primary antibody was removed and the cells were washed three times in 150 μL/well of PermWash. A total of 1:5000 αmouse/guinea pig-HRP secondary antibody (Cat#. A16104, ThermoFisher Scientific) was diluted in PermWash and added at 50 μL/well and rocked at room temperature for 1 h. The secondary antibody was removed and the cells were washed three times in 150 μL/well of PermWash. TrueBlue peroxidase substrate (SeraCare, Cat#. 5510-0030, Gaithersburg, MD, USA) was added at 30 μL/well, rocked for 15 min at room temperature, washed four times with deionized water, and dried on paper towel. This produced focus-forming units that were quantified on an ImmunoSpot ELISpot plate scanner (Cellular Technology Limited, Shaker Heights, OH, USA).

### 2.6. Lactate Dehydrogenase (LDH)-Based Cytotoxicity Assay

Cytotoxicity was assessed by measuring lactate dehydrogenase in the supernatant of iAs 24 h after mock infection or SARS-CoV-2 infection using the CytoTox96 non-radioactive cytotoxicity assay (Promega, Cat#. G1780, Madison, WI, USA) according to manufacturer instructions. Briefly, the cytotoxicity plate was incubated at 37 °C for 4 h to ensure accurate reading between target and effector cells. From each sample, 50 μL of supernatant were combined with 50 μL of Cyto Tox 96 Reagent (Promega, Cat#. G1780, Madison, WI, USA), covered with foil, and incubated on shaker at RT for 30 min and analyzed at 492 nm on the SoftMax Pro 7.0 machine (Molecular Devices, San Jose, CA, USA).

### 2.7. Western Blot Analysis and Antibodies

For Western Blotting, cells were lysed with radioimmunoprecipitation assay (RIPA) buffer (Cat. #AAJ63306 AK, Fisher Scientific, Hampton, NH, USA) and total protein content was estimated by bicinchoninic acid assay (BCA) on the SoftMax Pro 7.0 machine (Molecular Devices, San Jose, CA, USA). Five µg of total cell lysate was separated on a 4–20% SDS-PAGE, transferred on a polyvinylidene fluoride (PVDF) membrane, blocked with Intercept (TBS) blocking buffer (Cat. #927-60001, Li-Cor, Lincoln, NE, USA) for 1 h, and incubated with primary antibody for 1 h at room temperature (RT) with either β-catenin (1:500; Rabbit, Cell Signaling, Cat. #9582, USA) or β-actin (1:10,000, Mouse, Abcam, Cat. #ab6276, Cambridge, UK) overnight at 4 °C in Intercept (TBS) blocking buffer (Li-Cor Biosciences, Lincoln, NE, USA) and 0.2% Tween 20 (T20) (Cat #. J63314.K3, ThermoFisher Scientific, Waltham, MA, USA). Membranes were washed extensively in TBS-T20 and incubated in secondary antibody conjugated to horseradish peroxidase (HRP) IRDYE 700 nm or 800 nm (1:20,000, Rabbit or Mouse, Li-Cor Biosciences, Lincoln, NE, USA) in Intercept (TBS) blocking buffer and 0.1% T20 for 1 h at RT. Membranes were again washed extensively in TBS-T20, quickly washed in 1× TBS, and analyzed on the Licor Odyssey System (Li-Cor, Lincoln, NE, USA). Quantification analysis was conducted using Fiji (ImageJ) Software version 2.3.0 using the Gel Plotting Macro. Composite images of IRDYE 700 nm and 800 nm fluorescence were split into individual channels (Supplementary Figure S4). Regions of interest with identical dimensions were drawn on each lane at identical vertical position and density plots were generated.

### 2.8. Human IP-10 (CXCL10) ELISA

Human IP-10 (CXCL10) ELISA kit (Cat. #KAC2361, Invitrogen, Waltham, MA, USA) was used to measure CXCL10 secretion according to the manufacturer’s protocol. Briefly, 50 μL standards, supernatant samples, or controls were added to the antibody-coated plate. A total of 50 μL biotinylated anti-IP-10 biotin conjugate solution was added to each standard, sample, or control, covered with a plate cover, and incubated at RT for 3 h. Wells were washed extensively with washing buffer. A total of 100 μL Streptavidin-HRP was added to each well, covered with plate cover, and incubated for 30 min at RT. Wells were again washed extensively with washing buffer. A total of 50 μL of Stabilized Chromogen was added to each well and incubated in the dark at RT for 30 min. A total of 100 μL Stop Solution was added to each well, tapped gently for mixing, and analyzed at 450 nm on the SoftMax Pro 7.0 machine (Molecular Devices, San Jose, CA, USA).

### 2.9. Statistical Analysis

Statistical analyses were performed using Graphpad Prism (V10.6.0). Data were first analyzed for normality (or lognormality) of distribution of data using the Kolmogorov–Smirnov test (or the Shapiro–Wilk test for small data sets). Normally distributed data were analyzed with unpaired two-way T test, incorporating Welch’s correction when variances were unequal between groups. Lognormal data was assessed with the one sample ratio *t*-test, or the Kruskal–Wallis test followed by Dunn’s multiple comparisons test. Alpha was set to 0.05.

## 3. Results

### 3.1. Astrocytes Have a Sustained SARS-CoV-2 Infection After 24 h

iAs from human iPSC have been previously described [81]. Cellular identification of iAs culture was confirmed by flow cytometry with intracellular staining for the astrocyte intermediate filament protein GFAP. By this method, the majority (~90%) of cells in the iAs cultures were identified as astrocytes (Figure 1).

**Figure 2:**
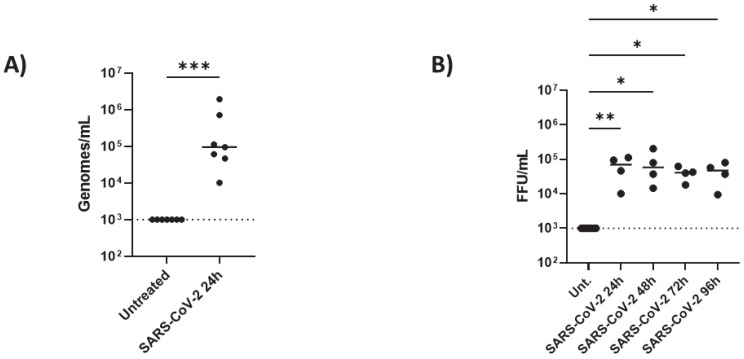
SARS-CoV viral infection in astrocytes: iAs at 90% confluency were infected with SARS-CoV-2 NY Iota strain for 24 h with the MOI = 3. (**A**) SARS-CoV-2 mRNA quantified in mock-infected or SARS-CoV-2-infected astrocytes at 24 h post infection. Kolmogorov–Smirnov test indicated lognormal distribution (KS distance 0.2334, *p* > 0.1000, alpha = 0.05). One sample ratio *t*-test demonstrated significant effect of infection on viral genomes (t = 7.268, df = 6, *p* = 0.0003). (**B**) SARS-CoV-2 infectious units in astrocyte supernatant over time were measured by focus-forming assay. Non-parametric Kruskal–Wallis one-way ANOVA demonstrated a significant effect of infection on FFU [h = 16.39 (5, 24); *p* = 0.0025]. Dunn’s multiple comparisons test demonstrated significant differences between uninfected and infected samples at 24 h (*p =* 0.0060), 48 h (*p =* 0.0108), 72 h (*p =* 0.0386), and 96 h (*p =* 0.0457). * *p* < 0.05, ** *p* < 0.01, *** *p* < 0.001. Data are presented from eight replicates for the mRNA and four replicates for the time course. Dashed line represents lower limit of detection. The statistical analysis of (**A**,**B**) is detailed in Tables S2 and S3.

Then, we determined whether iAs can be directly infected by SARS-CoV-2 in vitro by infecting them with SARS-CoV-2 strain B.1.526 (New York strain, Iota variant) for 24 h. After analyzing the viral titers and infectivity with qPCR, we found that iAs can be directly infected by SARS-CoV-2 (Figure 2A, *p*-value = 0.0006). This is consistent with previous studies of iAs being infected with SARS-CoV-2 [1,11,13,23]. Next, we measured infectious virus by focus-forming assay 24 h, 48 h, 72 h, and 96 h timepoints (Figure 2B). After 24 h post-infection, we detected approximately 1 × 10^5^ plaque-forming units (PFU) of virus in the supernatant (100,000 genome equivalents by qPCR) that was sustained up to 96 h (Figure 2B, *p*-value = 0.0060 for 24 h, *p*-value = 0.0108 for 48 h, *p*-value = 0.0386 for 72 h, and *p*-value = 0.0457 for 96 h). These data suggest a sustained secretion of viral particles achieving its equilibrium at 24 h post-infection in astrocytes.

### 3.2. SARS-CoV-2 Infection Inhibits β-Catenin (mRNA and Protein), Wnt3a, and Wnt10b While Inducing Wnt7b Transcription in Astrocytes

**Figure 3:**
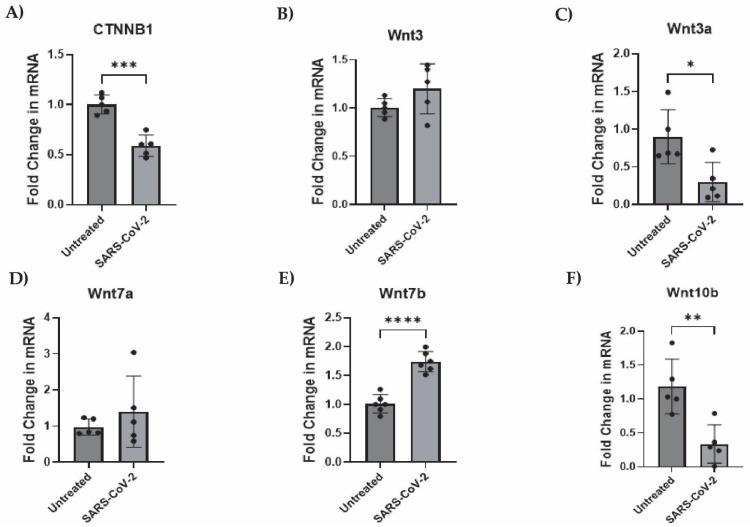
Wnts-3a and -10b and β-catenin transcription is suppressed while Wnt7b is stimulated by SARS-CoV-2 infection in astrocytes. iAs at 90% confluency were infected with SARS-CoV-2 NY Iota strain for 24 h with the MOI = 3. mRNA expression was measured by real-time PCR. Data is representative of 5–6 replicates. Error bars represent the SD. (**A**) Fold change in CTNNB1 (β-catenin) mRNA. Kolmogorov–Smirnov test demonstrated data are normally distributed (*p* > 0.1000). Unpaired *t*-test demonstrated significant effect of infection on CTNNB1 (*p* = 0.0002, t = 6.505, df = 8). (**B**) Fold change in Wnt3 mRNA. Kolmogorov–Smirnov test demonstrated data are normally distributed (*p* > 0.1000). Unpaired *t* test demonstrated no significant effect of infection (*p* = 0.1510, t = 1.588, df = 8). (**C**) Fold change in Wnt3a mRNA. Kolmogorov–Smirnov test demonstrated data are normally distributed (*p* > 0.1000). Unpaired *t*-test demonstrated significant effect of infection on Wnt3a (*p* = 0.0166, t = 3.020, df = 8). (**D**) Fold change in Wnt7a mRNA. Kolmogorov–Smirnov test demonstrated data are normally distributed (*p* > 0.1000). Unpaired *t* test with Welch’s correction for uneven variance demonstrated no significant effect of infection on Wnt7a (*p* = 0.3967, t = 0.9384, df = 4.399). (**E**) Fold change in Wnt7b mRNA. Kolmogorov–Smirnov test demonstrated data are normally distributed (*p* > 0.1000). Unpaired *t* test demonstrated significant effect of infection on Wnt7b (*p* < 0.0001, t = 7.518, df = 10). (**F**) Fold change in Wnt10b mRNA. Kolmogorov–Smirnov test demonstrated data are normally distributed (*p* > 0.1000). Unpaired *t* test demonstrated significant effect of infection on Wnt10b mRNA (*p* = 0.0049, t = 3.840, df = 8). * *p* < 0.05, ** *p* < 0.01, *** *p* < 0.001, **** *p* < 0.0001. The statistical analysis of (**A**–**F**) is detailed in Tables S4–S9.

**Figure 4:**
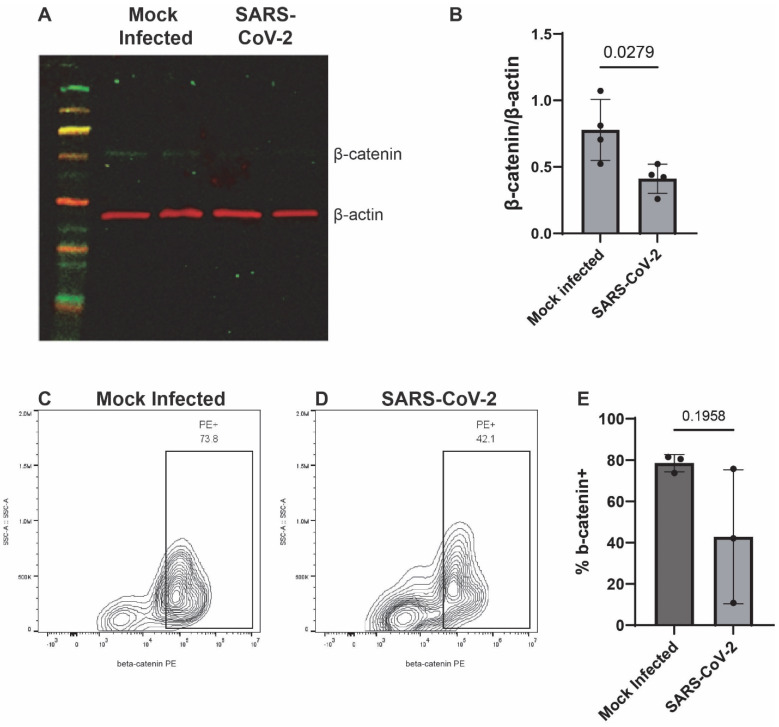
β-catenin is decreased in SARS-CoV-2-infected iAs. (**A**) iAs cell culture lysates were probed by Western Blot for β-catenin (~92 kDa) and actin (~42 kDa) 24 h after mock infection or SARS-CoV-2 infection. (**B**) Quantification of β-catenin in iAs protein lysates, normalized to actin. *p* < 0.05, Unpaired *t*-test. Shapiro’s test demonstrated normal distribution of data. (**C**,**D**) Flow cytometry was used to quantify β-catenin expression in live singlets in iAs cultures. (**E**) Quantification of flow cytometry data demonstrated a trend toward decreased β-catenin in SARS-CoV-2-infected iAs (mean, 42.9%) as compared to mock-infected iAs (mean, 78.6%), (*p* = 0.1958, Unpaired *t*-test with Welch’s correction for unequal variance). Shapiro’s test demonstrated normal distribution of data. PE: phycoerythrin. The statistical analysis of (**B**,**E**) is detailed in Tables S10 and S11.

To begin, we assessed the endogenous mRNA expression levels of components of the canonical and noncanonical Wnt pathways, particularly, CTNNB1 (β-catenin), Wnts-3, -3a, -5a, -7a, -7b, and -10b in iAs. qPCR analysis showed detectable levels of CTNNB1, Wnts-3, -3a, -5a, -7a, -7b, and -10b in iAs (Supplementary Figure S1A). With components of both Wnt canonical and noncanonical pathways being expressed, we focused on the canonical Wnt pathway since studies have shown it to be neuroprotective in neurocognitive and neurological disorders such as AD, HD, PD, HAND, and MS. Canonical Wnt signaling contains the transcriptional coactivator β-catenin, which binds to the TCF/LEF (consisting of TCF1, TCF3, TCF4, and LEF1) complex in the nucleus to regulate gene expression. In addition, astrocytes are known to robustly express the Wnt/β-catenin pathway [47,48,69]. To determine the effects of SARS-CoV-2 on the Wnt/β-catenin pathway, we infected iAs for 24 h and measured β-catenin, Wnts, and TCF/LEF mRNA by qPCR. β-catenin mRNA was reduced by ~50% (Figure 3A, *p*-value = 0.0002). β-catenin protein was significantly reduced ~50% by Western Blot (Figure 4A,B) and demonstrated a non-significant trend toward reduction by intracellular flow cytometry staining (Figure 4C–E). Wnt3a was significantly reduced by ~75% (Figure 3C, *p*-value = 0.0166), Wnt10b was significantly reduced by ~70% (Figure 3F, *p*-value = 0.0061) while Wnt7b was induced by ~2-fold (Figure 3E, *p*-value = <0.0001). Wnts-3 and -7 a were unaffected (Figure 3B,D). These results are potentially consistent with reduced neuronal regeneration (Wnt3a), neuronal survival and neurogenesis (Wnt10b), and increased BBB integrity (Wnt7b). Overall, these data indicate that multiple components in the Wnt/β-catenin pathway are dysregulated in astrocytes infected with SARS-CoV-2.

### 3.3. SARS-CoV-2 Infection Inhibits TCF3 Transcription in Astrocytes

**Figure 5:**
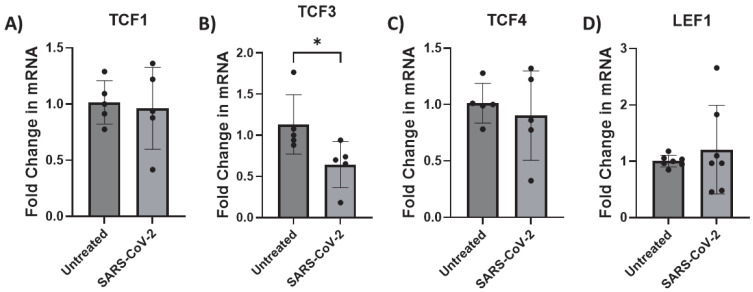
TCF3 transcription is downregulated by SARS-CoV-2 infection in astrocytes. iAs at 90% confluency were infected with SARS-CoV-2 NY Iota strain for 24 h with the MOI = 3. mRNA was measured by real-time PCR. Data represents 6–7 replicates. Error bars represent SD. (**A**) Fold change in TCF1 mRNA. Kolmogorov–Smirnov test demonstrated data are normally distributed (*p* > 0.1000). Unpaired *t* test demonstrated no significant effect of infection (*p* = 0.7859, t = 0.2809, df = 8). (**B**) Fold change in TCF3 mRNA. Kolmogorov–Smirnov test demonstrated data are not normally distributed (untreated: *p* = 0.0314, SARS-CoV-2: *p* > 0.1000). Mann–Whitney test demonstrated significant effect of infection on TCF3 mRNA (*p* = 0.0238). (**C**) Fold change in TCF4 mRNA. Kolmogorov–Smirnov test demonstrated data are normally distributed (*p* > 0.1000). Unpaired *t* test demonstrated no significant effect of infection on TCF4 mRNA (*p* = 0.5831, t = 0.5718, df = 8). (**D**) Fold change in LEF1 mRNA. Kolmogorov–Smirnov test demonstrated data are normally distributed (*p* > 0.1000). Unpaired *t* test with Welch’s correction demonstrated no significant effect of infection on LEF1 mRNA (*p* = 0.5205, t = 0.6809, df = 6.203). * *p* < 0.05. The statistical analysis of (**A**–**D**) is detailed in Tables S12–S15.

To define the mechanism by which β-catenin transcription is inhibited by SARS-CoV-2, we evaluated the impact of infection on transcription factors known to interact with β-catenin: TCF1, TCF3, TCF4, and LEF1. All were expressed in iAs, as consistent with other studies [68,75,81] (Supplementary Figure S1B). Then, we measured TCFs/LEF mRNA in SARS-CoV-2-infected astrocytes by qPCR. In this family of transcription factors, we observed that TCF3 was reduced by ~40% (Figure 5B, *p*-value = 0.0435). In contrast, TCFs-1, -4, and LEF1 were unaffected (Figure 5A,C,D). These data indicate that TCF3 is directly impacted by SARS-CoV-2 infection in astrocytes and is consistent with dysregulation of the Wnt/β-catenin pathway in consequence of infection.

### 3.4. SARS-CoV-2 Infection Activates Astrocytes by Inducing CXCL10 Transcription

**Figure 6:**
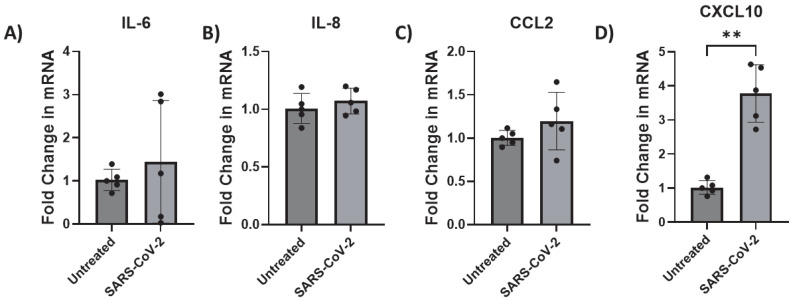
CXCL10 transcription is upregulated by SARS-CoV-2 infection in astrocytes. iAs at 90% confluency were infected with SARS-CoV-2 NY Iota strain MOI = 3 for 24 h. mRNA was measured by real-time PCR. Data represent five replicates. Error bars represent SD. (**A**) Fold change in IL-6 mRNA. Kolmogorov–Smirnov test demonstrated data are normally distributed (*p* > 0.1000). Unpaired *t* test with Welch’s correction demonstrated no significant effect of infection on IL-6 mRNA (*p* = 0.5454, t = 0.6566, df = 4.243). (**B**) Fold change in IL-8 mRNA. Kolmogorov–Smirnov test demonstrated data are normally distributed (*p* > 0.1000). Unpaired *t* test with Welch’s correction demonstrated no significant effect of infection (*p* = 0.4228, t = 0.8448, df = 8). (**C**) Fold change in CCL2 mRNA. Kolmogorov–Smirnov test demonstrated data are normally distributed (*p* > 0.1000). Unpaired *t* test with Welch’s correction demonstrated no significant effect (*p* = 0.2653, t = 1.270, df = 4.532). (**D**) Fold change in CXCL10 mRNA. Kolmogorov–Smirnov test demonstrated data are normally distributed (*p* > 0.1000). Unpaired *t* test with Welch’s correction demonstrated significant effect of infection on CXCL10 (*p* = 0.0014, t = 7.103, df = 4.463). ** *p* < 0.01. The statistical analysis of (**A**–**D**) is detailed in Tables S16–S19.

**Figure 7:**
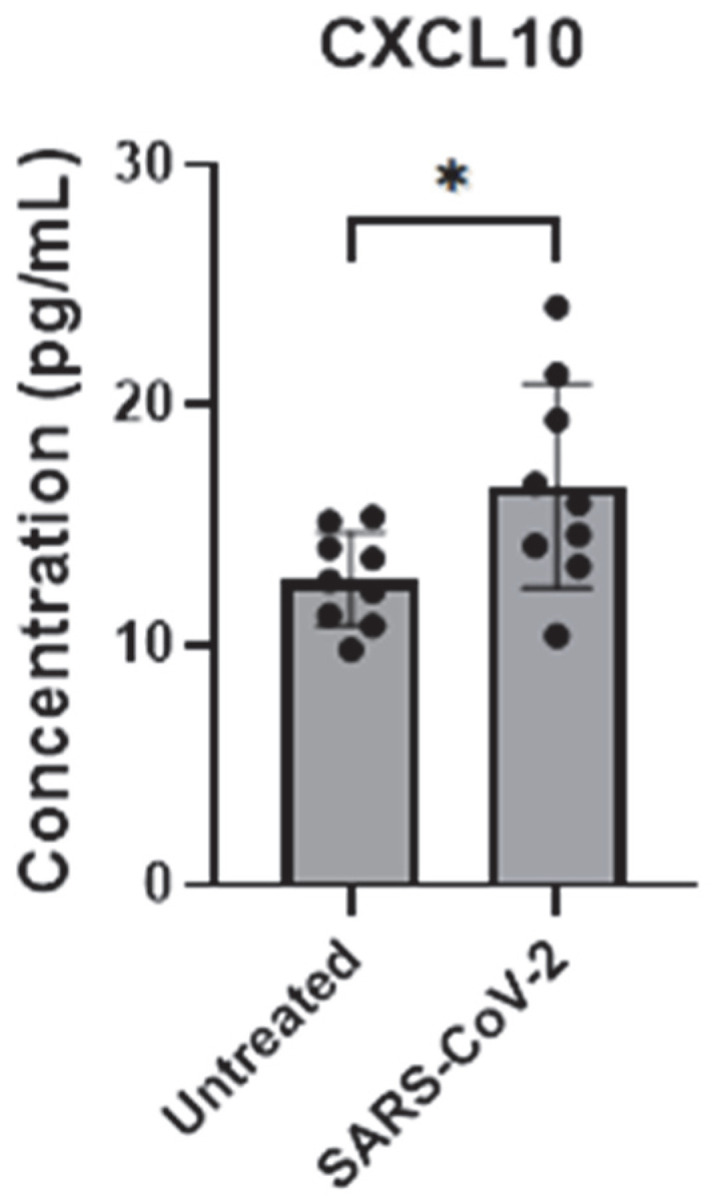
CXCL10 secretion is upregulated by SARS-CoV-2 infection in astrocytes. iAs at 90% confluency were infected with SARS-CoV-2 NY Iota strain MOI = 3 for 24 h. CXCL10 in supernatant was measured by ELISA. Data represent nine replicates. Error bars represent SD. Kolmogorov–Smirnov test demonstrated data are normally distributed. Unpaired *t*-test with Welch’s correction demonstrated significant effect of infection on CXCL10 in the supernatant (*p* = 0.0307, t = 2.474, df = 11.14). * *p* < 0.05. The statistical analysis of Figure 7 is detailed in Table S20.

Here, we assessed the endogenous mRNA expression levels of selected inflammatory factors (IL-6, IL-8, CCL2, and CXCL10) in iAs. qPCR analysis showed detectable expression of these inflammatory factors with Ct values less than 32 (Supplementary Figure S2). To determine which and whether SARS-CoV-2 infection affects cytokine IL-6 and chemokines (IL-8, CCL2 (also MCP-1), and CXCL10), we investigated their mRNA expression in SARS-CoV-2-infected astrocytes by qPCR. We found that while IL-6, IL-8, and CCL2 were unaffected (Figure 6A–C), CXCL10 mRNA was significantly induced ~4-fold (Figure 6D, *p*-value = 0.0014). CXCL10 protein was upregulated about 1.5-fold (Figure 7, *p*-value = 0.0307) by ELISA in the supernatant of mock- and SARS-CoV-2-infected astrocytes. These data show that SARS-CoV-2 infection can activate astrocytes and is indicative of SARS-CoV-2 infection and possibly signals in a type II interferon-dependent manner.

### 3.5. SARS-CoV-2 Does Not Induce Significant Cytotoxicity in Astrocytes

**Figure 8:**
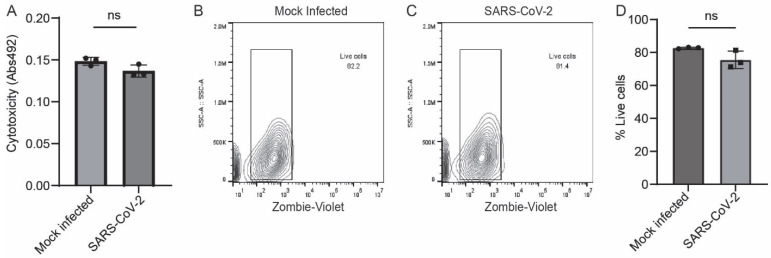
No significant cell death in SARS-CoV-2-infected iAs. (**A**) Cytotoxicity was measured by colorimetric assay for detection of lactate dehydrogenase (LDH) in the supernatant of iAs 24 h after mock or SARS-CoV-2 infection. Data is representative of three replicates. Error bars represent the SD. Shapiro–Wilk test indicated that data were normally distributed. Unpaired two-way T test revealed no significant difference in LDH secretion from mock and SARS-CoV-2-infected iAs (*p* = 0.7950, t = 2.777, df = 4). (**B**,**C**) Flow cytometry plots for Zombie Live/Dead demonstrate similar distribution of vital dye intensity 24 h after mock and SARS-CoV-2 infection. (**D**) Quantification of vital dye intensity by flow cytometry demonstrates no significant difference in cell death 24 h after infection with mock or SARS-CoV-2 infection in iAs. Data is representative of three replicates. Error bars represent the SD. Shapiro–Wilk test indicated that data were normally distributed. Unpaired two-way T test revealed no significant differences in vital dye exclusion (*p* = 0.0776, t = 2.361, df = 4). The statistical analysis of (**A**,**D**) is detailed in Tables S21 and S22.

To assess whether SARS-CoV-2 infection leads to cell death in astrocytes, we measured the levels of lactate dehydrogenase in the supernatant of SARS-CoV-2-infected and mock-infected astrocytes. Lactate dehydrogenase is a cytosolic enzyme released from the cell upon cell lysis, which is indicative of apoptosis. We found no differences in lactate dehydrogenase secretion levels between SARS-CoV-2-infected and mock-infected astrocytes (Figure 8A). Furthermore, we measured cell death by vital dye exclusion in a flow cytometry-based assay (Figure 8B,C). We observed no significant difference in cell viability by vital dye exclusion (Figure 8D). Overall, these data show that the changes in iAs Wnt/β-catenin and inflammatory signaling in response to SARS-CoV-2 infection are unlikely to be secondary to astrocyte cell death.

## 4. Discussion

The neuropathogenesis of SARS-CoV-2 infection is incompletely understood. Viral infection of CNS cells could contribute to neurodegeneration. Emerging studies show CNS cells including astrocytes can be infected by the SARS-CoV-2 virus [1,11,21,23]. Consistent with this, we report that iAs are susceptible to infection in vitro; however production of new infectious particles is mostly limited to the first 24 hours post infection. The extent to which CNS cells are infected with SARS-CoV-2 in vivo is unclear. It is possible that the infection of CNS cells has been underestimated in vivo because of the lack of ongoing active production of viral proteins. Our data is consistent with the possibility that astrocyte infection with SARS-CoV-2 could be more extensive than what is currently known and therefore may have an underappreciated role in long term neuropathology after COVID-19. Moreover, comorbidities such as prior viral infection or immunosuppression upregulate expression of SARS-CoV-2 receptors [21]. Therefore, greater CNS infection with SARS-CoV-2 could contribute to increased COVID-19/Long COVID neurological disability in patients with immunologic or neurologic comorbidities.

Our study has limitations. Our study focused on Wnt/β-catenin and inflammatory pathways; we have not probed all the cell signaling pathways that are likely to impact SARS-CoV-2 neuroinflammation. Our study did not address the interactions between astrocytes and other cells of the CNS. For example, we have not tested the effect of the infected astrocyte secretome on microglia, endothelial cells, or other cells. Likewise, these studies were not designed to test the consequences to astrocytes of microglial infection or exposure to viral components. In vivo, the close interactions between cells of the neurovascular unit are expected to modify all aspects of the neuroimmunological response to infection. We have not tested the potential combinatorial effect to astrocytes of exposure to SARS-CoV-2 virus plus inflammatory factors such as interferons or interleukins that are elevated in the blood and brain during infection.

Astrocytes have several functions which include secretion of cytokines/chemokines and maintaining BBB integrity. Previous studies have shown that endogenous levels of cytokines and chemokines are expressed with Ct values being below 33 in astrocytes [68,75]. BBB integrity is promoted by astrocyte production of canonical Wnt ligands [28]. Since BBB integrity is disrupted and CXCL10 levels are induced in COVID-19/Long COVID, we assessed the impact of SARS-CoV-2 direct infection on the Wnt/β-catenin pathway. As expected, we observed that Wnt canonical ligands Wnt3, Wnt3 a, Wnt7 a, Wnt7b, and Wnt10b, and noncanonical Wnt5 a, are all expressed in astrocytes. In addition, the entire TCF/LEF complex is expressed in astrocytes. A main finding of our study is that Wnt3a is downregulated by direct infection. Wnt3a promotes BBB integrity and neurogenesis [28,30] and its downregulation is associated with BBB leakage and neuroinflammation in traumatic brain injury [82] and ischemic stroke [83]. Consistently, virally induced downregulation of Wnt3a could contribute to BBB permeability in COVID-19/Long COVID. Wnt10b is known to be involved in axonal regeneration [84] and neuronal development [85] in the brain. One study shows that Wnt10b is upregulated in astrocytes upon HIV infection, suggesting that Wnt10b could have some anti-viral activity in astrocytes [86]. In contrast, however, we also observed upregulation of Wnt7b, another canonical Wnt ligand secreted by astrocytes with critical roles in astrocytic development, maintaining the BBB integrity, and BBB repair after ischemic stroke [61,87]. Upregulation of Wnt7b could be consistent with a neuroprotective response. Our study supports that in response to direct infection, astrocytes decrease anti-inflammatory and increase proinflammatory pathways. Furthermore, we observed that SARS-CoV-2 downregulates β-catenin and TCF3, suggestive of suppressed intracellular Wnt/β-catenin signaling activity and Wnt10b transcription in iAs.

We also observed that SARS-CoV-2 infection of astrocytes induced the inflammatory chemokine CXCL10, like previous reports [1,23]. This is consistent with reports of elevated CXCL10 in the brains of COVID-19 patients [88,89,90]. Elevated levels of CXCL10 are also found in neurodegenerative disorders such as MS [91,92,93], AD [94], and HAND [95,96,97]. Thus, production of proinflammatory chemokines in a type II interferon-dependent manner in astrocytes is another potential mechanism of SARS-CoV-2 induced neuroinflammation.

This study shows that SARS-CoV-2 infection affects some components of the canonical Wnt/β-catenin signaling pathway in astrocytes. SARS-CoV-2 downregulates β-catenin, TCF3, Wnt3a, and Wnt10b while simultaneously inducing Wnt7b and CXCL10. These data are consistent with a complex cellular response to infection (Supplementary Figure S3). Future studies will be required to determine the extent to which astrocyte infection with SARS-CoV-2 in vivo contributes neuroinflammation and neurological impairment in COVID-19/Long COVID. Recent studies have shown in vivo evidence that the brain is subject to neuroinvasion and neuroinflammation due to SARS-CoV-2 infection in the K18-hACE2 mouse model [98,99,100,101,102]. Our study explores the Wnt/β-catenin pathway as a mechanism that can possibly lead to therapeutic studies of neuroinflammation and neurological impairments in COVID-19/Long COVID.

## Data Availability

Data are contained within the article.

## Supplementary Materials

The following supporting information can be downloaded at https://www.mdpi.com/article/10.3390/pathogens14100994/s1; Figure S1: Endogenous expression of Wnt signaling components in iAs; Figure S2: Endogenous expression of inflammatory genes and type I interferons in iAs; Figure S3: SARS-CoV-2 direct infection of iAs in vitro alters multiple homeostatic and inflammatory factors. Figure S4: Raw data and quantification for Western blots depicted in Figure 4. Table S1: iAs cultures contain astrocytes as assessed by flow cytometry for GFAP; Table S2: SARS-CoV-2 Viral infection in astrocytes: genomes/ml; Table S3: SARS-CoV-2 infectious units in astrocyte supernatant over time; Table S4: Ctnnb1 mRNA fold change; Table S5: Wnt3 mRNA; Table S6: Fold change in Wnt3a mRNA; Table S7: Fold change in Wnt7a mRNA; Table S8: Fold change in Wn7b mRNA; Table S9: Fold change in Wnt10b mRNA; Table S10: Fold change in β-catenin protein as analyzed by Western Blot; Table S11: Cells positive for β-catenin protein as analyzed by intracellular staining flow cytometry; Table S12: Fold change in TCF1 mRNA; Table S13: Fold change in TCF3 mRNA; Table S14: Fold change in TCF4 mRNA; Table S15: Fold change in LEF1 mRNA; Table S16: IL6 mRNA; Table S17: IL8 mRNA; Table S18: Fold change in CCL2 mRNA; Table S19: Fold change in CXCL10 mRNA; Table S20: CXCL10 ELISA; Table S21: Cytotoxicity in infected astrocytes by assay for LDH secretion; Table S22: Cell death in infected astrocytes by flow cytometry staining with Zombie violet.

## References

[1] Kong W., Montano M., Corley M.J., Helmy E., Kobayashi H., Kinisu M., Suryawanshi R., Luo X., Royer L.A., Roan N.R. (2022). Neuropilin-1 Mediates SARS-CoV-2 Infection of Astrocytes in Brain Organoids, Inducing Inflammation Leading to Dysfunction and Death of Neurons. mBio.

[2] Green R., Mayilsamy K., McGill A.R., Martinez T.E., Chandran B., Blair L.J., Bickford P.C., Mohapatra S.S., Mohapatra S. (2022). SARS-CoV-2 infection increases the gene expression profile for Alzheimer’s disease risk. Mol. Ther. Methods Clin. Dev..

[3] Rutkai I., Mayer M.G., Hellmers L.M., Ning B., Huang Z., Monjure C.J., Coyne C., Silvestri R., Golden N., Hensley K. (2022). Neuropathology and virus in brain of SARS-CoV-2 infected non-human primates. Nat. Commun..

[4] Taquet M., Geddes J.R., Husain M., Luciano S., Harrison P.J. (2021). 6-month neurological and psychiatric outcomes in 236 379 survivors of COVID-19: A retrospective cohort study using electronic health records. Lancet Psychiatry.

[5] Xie Y., Bowe B., Al-Aly Z. (2021). Burdens of post-acute sequelae of COVID-19 by severity of acute infection, demographics and health status. Nat. Commun..

[6] Davis H.E., McCorkell L., Vogel J.M., Topol E.J. (2023). Long COVID: Major findings, mechanisms and recommendations. Nat. Rev. Microbiol..

[7] Stein S.R., Ramelli S.C., Grazioli A., Chung J.-Y., Singh M., Yinda C.K., Winkler C.W., Sun J., Dickey J.M., Ylaya K. (2022). SARS-CoV-2 infection and persistence in the human body and brain at autopsy. Nature.

[8] Matschke J., Lütgehetmann M., Hagel C., Sperhake J.P., Schröder A.S., Edler C., Mushumba H., Fitzek A., Allweiss L., Dandri M. (2020). Neuropathology of patients with COVID-19 in Germany: A post-mortem case series. Lancet Neurol..

[9] Granholm A.-C.E., Englund E., Gilmore A., Head E., Yong W.H., Perez S.E., Guzman S.J., Hamlett E.D., Mufson E.J. (2024). Neuropathological findings in Down syndrome, Alzheimer’s disease and control patients with and without SARS-CoV-2: Preliminary findings. Acta Neuropathol..

[10] Motta C.S., Torices S., da Rosa B.G., Marcos A.C., Alvarez-Rosa L., Siqueira M., Moreno-Rodriguez T., Matos A.D.R., Caetano B.C., Martins J. (2023). Human Brain Microvascular Endothelial Cells Exposure to SARS-CoV-2 Leads to Inflammatory Activation through NF-κB Non-Canonical Pathway and Mitochondrial Remodeling. Viruses.

[11] Andrews M.G., Mukhtar T., Eze U.C., Simoneau C.R., Ross J., Parikshak N., Wang S., Zhou L., Koontz M., Velmeshev D. (2022). Tropism of SARS-CoV-2 for human cortical astrocytes. Proc. Natl. Acad. Sci. USA.

[12] Thakur K.T., Miller E.H., Glendinning M.D., Al-Dalahmah O., Banu M.A., Boehme A.K., Boubour A.L., Bruce S.S., Chong A.M., Claassen J. (2021). COVID-19 neuropathology at Columbia University Irving Medical Center/New York Presbyterian Hospital. Brain.

[13] Crunfli F., Carregari V.C., Veras F.P., Vendramini P.H., Valença A.G.F., Antunes A.S.L.M., Brandão-Teles C., Zuccoli G.d.S., Reis-de-Oliveira G., Silva-Costa L.C. (2022). Morphological, cellular and molecular basis of brain infection in COVID-19 patients. Proc. Natl. Acad. Sci. USA.

[14] Khan M., Clijsters M., Choi S., Backaert W., Claerhout M., Couvreur F., Van Breda L., Bourgeois F., Speleman K., Klein S. (2022). Anatomical barriers against SARS-CoV-2 neuroinvasion at vulnerable interfaces visualized in deceased COVID-19 patients. Neuron.

[15] Khan M., Yoo S.J., Clijsters M., Backaert W., Vanstapel A., Speleman K., Lietaer C., Choi S., Hether T.D., Marcelis L. (2021). Visualizing in deceased COVID-19 patients how SARS-CoV-2 attacks the respiratory and olfactory mucosae but spares the olfactory bulb. Cell.

[16] Haverty R., McCormack J., Evans C., Purves K., O’Reilly S., Gautier V., Rochfort K., Fabre A., Fletcher N.F. (2024). SARS-CoV-2 infects neurons, astrocytes, choroid plexus epithelial cells and pericytes of the human central nervous system in vitro. J. Gen. Virol..

[17] Colinet M., Chiver I., Bonafina A., Masset G., Almansa D., Di Valentin E., Twizere J.C., Nguyen L., Espuny-Camacho I. (2025). SARS-CoV-2 infection triggers inflammatory conditions and astrogliosis-related gene expression in long-term human cortical organoids. Stem Cells.

[18] Wang C., Zhang M., Garcia G., Tian E., Cui Q., Chen X., Sun G., Wang J., Arumugaswami V., Shi Y. (2021). ApoE-Isoform-Dependent SARS-CoV-2 Neurotropism and Cellular Response. Cell Stem Cell.

[19] Albornoz E.A., Amarilla A.A., Modhiran N., Parker S., Li X.X., Wijesundara D.K., Aguado J., Zamora A.P., McMillan C.L., Liang B. (2023). SARS-CoV-2 drives NLRP3 inflammasome activation in human microglia through spike protein. Mol. Psychiatry.

[20] Jeong G.U., Lyu J., Kim K.-D., Chung Y.C., Yoon G.Y., Lee S., Hwang I., Shin W.-H., Ko J., Lee J.-Y. (2022). SARS-CoV-2 Infection of Microglia Elicits Proinflammatory Activation and Apoptotic Cell Death. Microbiol. Spectr..

[21] Torices S., Cabrera R., Stangis M., Naranjo O., Fattakhov N., Teglas T., Adesse D., Toborek M. (2021). Expression of SARS-CoV-2-related receptors in cells of the neurovascular unit: Implications for HIV-1 infection. J. Neuroinflamm..

[22] Yamada S., Hashita T., Yanagida S., Sato H., Yasuhiko Y., Okabe K., Noda T., Nishida M., Matsunaga T., Kanda Y. (2024). SARS-CoV-2 causes dysfunction in human iPSC-derived brain microvascular endothelial cells potentially by modulating the Wnt signaling pathway. Fluids Barriers CNS.

[23] Chaves J.C., Milton L.A., Stewart R., Senapati T., Rantanen L.M., Wasielewska J.M., Lee S., Hernández D., McInnes L., Quek H. (2024). Differential Cytokine Responses of APOE3 and APOE4 Blood–brain Barrier Cell Types to SARS-CoV-2 Spike Proteins. J. Neuroimmune Pharmacol..

[24] Potokar M., Zorec R., Jorgačevski J. (2023). Astrocytes Are a Key Target for Neurotropic Viral Infection. Cells.

[25] Greene C., Connolly R., Brennan D., Laffan A., O’Keeffe E., Zaporojan L., O’Callaghan J., Thomson B., Connolly E., Argue R. (2024). Blood-brain barrier disruption and sustained systemic inflammation in individuals with long COVID-associated cognitive impairment. Nat. Neurosci..

[26] Bayat A.H., Azimi H., Hassani Moghaddam M., Ebrahimi V., Fathi M., Vakili K., Mahmoudiasl G.R., Forouzesh M., Boroujeni M.E., Nariman Z. (2022). COVID-19 causes neuronal degeneration and reduces neurogenesis in human hippocampus. Apoptosis Int. J. Program. Cell Death.

[27] Stępień T., Tarka S., Chmura N., Grzegorczyk M., Acewicz A., Felczak P., Wierzba-Bobrowicz T. (2023). Influence of SARS-CoV-2 on Adult Human Neurogenesis. Cells.

[28] Guérit S., Fidan E., Macas J., Czupalla C.J., Figueiredo R., Vijikumar A., Yalcin B.H., Thom S., Winter P., Gerhardt H. (2021). Astrocyte-derived Wnt growth factors are required for endothelial blood-brain barrier maintenance. Prog. Neurobiol..

[29] Hussain B., Fang C., Huang X., Feng Z., Yao Y., Wang Y., Chang J. (2022). Endothelial β-Catenin Deficiency Causes Blood-Brain Barrier Breakdown via Enhancing the Paracellular and Transcellular Permeability. Front. Mol. Neurosci..

[30] Yue J., Mo L., Zeng G., Ma P., Zhang X., Peng Y., Zhang X., Zhou Y., Jiang Y., Huang N. (2025). Inhibition of neutrophil extracellular traps alleviates blood-brain barrier disruption and cognitive dysfunction via Wnt3/β-catenin/TCF4 signaling in sepsis-associated encephalopathy. J. Neuroinflamm..

[31] Sebo D.J., Ali I., Fetsko A.R., Trimbach A.A., Taylor M.R. (2025). Activation of Wnt/β-catenin in neural progenitor cells regulates blood-brain barrier development and promotes neuroinflammation. Sci. Rep..

[32] Huang X., Wei P., Fang C., Yu M., Yang S., Qiu L., Wang Y., Xu A., Hoo R.L.C., Chang J. (2024). Compromised endothelial Wnt/β-catenin signaling mediates the blood-brain barrier disruption and leads to neuroinflammation in endotoxemia. J. Neuroinflamm..

[33] Daneman R., Agalliu D., Zhou L., Kuhnert F., Kuo C.J., Barres B.A. (2009). Wnt/β-catenin signaling is required for CNS, but not non-CNS, angiogenesis. Proc. Natl. Acad. Sci. USA.

[34] Martin M., Vermeiren S., Bostaille N., Eubelen M., Spitzer D., Vermeersch M., Profaci C.P., Pozuelo E., Toussay X., Raman-Nair J. (2022). Engineered Wnt ligands enable blood-brain barrier repair in neurological disorders. Science.

[35] Benz F., Wichitnaowarat V., Lehmann M., Germano R.F., Mihova D., Macas J., Adams R.H., Taketo M.M., Plate K.H., Guérit S. (2019). Low wnt/β-catenin signaling determines leaky vessels in the subfornical organ and affects water homeostasis in mice. eLife.

[36] Chang J., Mancuso M.R., Maier C., Liang X., Yuki K., Yang L., Kwong J.W., Wang J., Rao V., Vallon M. (2017). Gpr124 is essential for blood-brain barrier integrity in central nervous system disease. Nat. Med..

[37] Cho C., Smallwood P.M., Nathans J. (2017). Reck and Gpr124 Are Essential Receptor Cofactors for Wnt7a/Wnt7b-Specific Signaling in Mammalian CNS Angiogenesis and Blood-Brain Barrier Regulation. Neuron.

[38] Choi E.Y., Park H.H., Kim H., Kim H.N., Kim I., Jeon S., Kim W., Bae J.-S., Lee W. (2020). Wnt5a and Wnt11 as acute respiratory distress syndrome biomarkers for SARS-CoV-2 patients. Eur. Respir. J..

[39] Xu Z., Elaish M., Wong C.P., Hassan B.B., Lopez-Orozco J., Felix-Lopez A., Ogando N.S., Nagata L., Mahal L.K., Kumar A. (2024). The Wnt/β-catenin pathway is important for replication of SARS-CoV-2 and other pathogenic RNA viruses. npj Viruses.

[40] Trevino T.N., Fogel A.B., Otkiran G., Niladhuri S.B., Sanborn M.A., Class J., Almousawi A.A., Vanhollebeke B., Tai L.M., Rehman J. (2024). Engineered Wnt7a ligands rescue blood–brain barrier and cognitive deficits in a COVID-19 mouse model. Brain.

[41] Mayer M.G., Fischer T. (2025). Shared Mechanisms of Blood-Brain Barrier Dysfunction and Neuroinflammation in Coronavirus Disease 2019 and Alzheimer Disease. Am. J. Pathol..

[42] Kalani M.Y., Cheshier S.H., Cord B.J., Bababeygy S.R., Vogel H., Weissman I.L., Palmer T.D., Nusse R. (2008). Wnt-mediated self-renewal of neural stem/progenitor cells. Proc. Natl. Acad. Sci. USA.

[43] Lie D.C., Colamarino S.A., Song H.J., Désiré L., Mira H., Consiglio A., Lein E.S., Jessberger S., Lansford H., Dearie A.R. (2005). Wnt signalling regulates adult hippocampal neurogenesis. Nature.

[44] Nishihara H., Perriot S., Gastfriend B.D., Steinfort M., Cibien C., Soldati S., Matsuo K., Guimbal S., Mathias A., Palecek S.P. (2022). Intrinsic blood-brain barrier dysfunction contributes to multiple sclerosis pathogenesis. Brain.

[45] Gastfriend B.D., Nishihara H., Canfield S.G., Foreman K.L., Engelhardt B., Palecek S.P., Shusta E.V. (2021). Wnt signaling mediates acquisition of blood-brain barrier properties in naïve endothelium derived from human pluripotent stem cells. eLife.

[46] Laksitorini M.D., Yathindranath V., Xiong W., Hombach-Klonisch S., Miller D.W. (2019). Modulation of Wnt/β-catenin signaling promotes blood-brain barrier phenotype in cultured brain endothelial cells. Sci. Rep..

[47] Al-Harthi L. (2012). Interplay between Wnt/β-catenin signaling and HIV: Virologic and biologic consequences in the CNS. J. Neuroimmune Pharmacol. Off. J. Soc. NeuroImmune Pharmacol..

[48] Al-Harthi L. (2012). Wnt/beta-catenin and its diverse physiological cell signaling pathways in neurodegenerative and neuropsychiatric disorders. J. Neuroimmune Pharmacol. Off. J. Soc. NeuroImmune Pharmacol..

[49] Aljawai Y., Richards M.H., Seaton M.S., Narasipura S.D., Al-Harthi L. (2014). β-Catenin/TCF-4 signaling regulates susceptibility of macrophages and resistance of monocytes to HIV-1 productive infection. Curr. HIV Res..

[50] Henderson L.J., Al-Harthi L. (2011). Role of β-catenin/TCF-4 signaling in HIV replication and pathogenesis: Insights to informing novel anti-HIV molecular therapeutics. J. Neuroimmune Pharmacol. Off. J. Soc. NeuroImmune Pharmacol..

[51] Narasipura S.D., Henderson L.J., Fu S.W., Chen L., Kashanchi F., Al-Harthi L. (2012). Role of β-catenin and TCF/LEF family members in transcriptional activity of HIV in astrocytes. J. Virol..

[52] Jimenez O.A., Narasipura S.D., Barbian H.J., Albalawi Y.A., Seaton M.S., Robinson K.F., Al-Harthi L. (2021). β-Catenin Restricts Zika Virus Internalization by Downregulating Axl. J. Virol..

[53] Lengfeld J.E., Lutz S.E., Smith J.R., Diaconu C., Scott C., Kofman S.B., Choi C., Walsh C.M., Raine C.S., Agalliu I. (2017). Endothelial Wnt/β-catenin signaling reduces immune cell infiltration in multiple sclerosis. Proc. Natl. Acad. Sci. USA.

[54] Ponath G., Park C., Pitt D. (2018). The Role of Astrocytes in Multiple Sclerosis. Front. Immunol..

[55] Tapia-Rojas C., Inestrosa N.C. (2018). Loss of canonical Wnt signaling is involved in the pathogenesis of Alzheimer’s disease. Neural Regen. Res..

[56] Jia L., Piña-Crespo J., Li Y. (2019). Restoring Wnt/β-catenin signaling is a promising therapeutic strategy for Alzheimer’s disease. Mol. Brain.

[57] Inestrosa N.C., Montecinos-Oliva C., Fuenzalida M. (2012). Wnt signaling: Role in Alzheimer disease and schizophrenia. J. Neuroimmune Pharmacol..

[58] Berwick D.C., Harvey K. (2012). The importance of Wnt signaling for neurodegeneration in Parkinson’s Disease. Biochem. Soc. Trans..

[59] L’episcopo F., Tirolo C., Caniglia S., Testa N., Morale M.C., Serapide M.F., Pluchino S., Marchetti B. (2014). Targeting Wnt signaling at the neuroimmune interface for dopaminergic neuroprotection/repair in Parkinson’s disease. J. Mol. Cell Biol..

[60] Lim R.G., Quan C., Reyes-Ortiz A.M., Lutz S.E., Kedaigle A.J., Gipson T.A., Wu J., Vatine G.D., Stocksdale J., Casale M.S. (2017). Huntington’s disease iPSC-derived brain microvascular endothelial cells reveal WNT-mediated angiogenic and blood-brain barrier deficits. Cell Rep..

[61] Song S., Huang H., Guan X., Fiesler V., Bhuiyan M.I.H., Liu R., Jalali S., Hasan M.N., Tai A.K., Chattopadhyay A. (2021). Activation of endothelial Wnt/β-catenin signaling by protective astrocytes repairs BBB damage in ischemic stroke. Prog. Neurobiol..

[62] Qu X., Yang R., Tan C., Chen H., Wang X. (2025). Astrocytes-Secreted WNT5B Disrupts the Blood-Brain Barrier Via ROR1/JNK/c-JUN Cascade During Meningitic Escherichia Coli Infection. Mol. Neurobiol..

[63] Okamoto M., Inoue K., Iwamura H., Terashima K., Soya H., Asashima M., Kuwabara T. (2011). Reduction in paracrine Wnt3 factors during aging causes impaired adult neurogenesis. FASEB J. Off. Publ. Fed. Am. Soc. Exp. Biol..

[64] Carroll-Anzinger D., Al-Harthi L. (2006). Gamma interferon primes productive human immunodeficiency virus infection in astrocytes. J. Virol..

[65] Li W., Henderson L.J., Major E.O., Al-Harthi L. (2011). IFN-γ mediates enhancement of HIV replication in astrocytes by inducing an antagonist of the β-catenin pathway (DKK1) in a STAT 3-dependent manner. J. Immunol..

[66] Henderson L.J., Sharma A., Monaco M.C., Major E.O., Al-Harthi L. (2012). Human immunodeficiency virus type 1 (HIV-1) transactivator of transcription through its intact core and cysteine-rich domains inhibits Wnt/β-catenin signaling in astrocytes: Relevance to HIV neuropathogenesis. J. Neurosci. Off. J. Soc. Neurosci..

[67] Lutgen V., Narasipura S.D., Barbian H.J., Richards M., Wallace J., Razmpour R., Buzhdygan T., Ramirez S.H., Prevedel L., Eugenin E.A. (2020). HIV infects astrocytes in vivo and egresses from the brain to the periphery. PLoS Pathog..

[68] Robinson K.F., Narasipura S.D., Wallace J., Ritz E.M., Al-Harthi L. (2020). β-Catenin and TCFs/LEF signaling discordantly regulate IL-6 expression in astrocytes. Cell Commun. Signal..

[69] Lutgen V., Narasipura S.D., Sharma A., Min S., Al-Harthi L. (2016). β-Catenin signaling positively regulates glutamate uptake and metabolism in astrocytes. J. Neuroinflamm..

[70] Carroll-Anzinger D., Kumar A., Adarichev V., Kashanchi F., Al-Harthi L. (2007). Human immunodeficiency virus-restricted replication in astrocytes and the ability of gamma interferon to modulate this restriction are regulated by a downstream effector of the Wnt signaling pathway. J. Virol..

[71] Henderson L.J., Narasipura S.D., Adarichev V., Kashanchi F., Al-Harthi L. (2012). Identification of novel T cell factor 4 (TCF-4) binding sites on the HIV long terminal repeat which associate with TCF-4, β-catenin, and SMAR1 to repress HIV transcription. J. Virol..

[72] Erta M., Quintana A., Hidalgo J. (2012). Interleukin-6, a major cytokine in the central nervous system. Int. J. Biol. Sci..

[73] Mauer J., Denson J.L., Brüning J.C. (2015). Versatile functions for IL-6 in metabolism and cancer. Trends Immunol..

[74] Rothaug M., Becker-Pauly C., Rose-John S. (2016). The role of interleukin-6 signaling in nervous tissue. Biochim. Biophys. Acta (BBA)-Mol. Cell Res..

[75] Robinson K.F., Narasipura S.D., Wallace J., Ritz E.M., Al-Harthi L. (2020). Negative regulation of IL-8 in human astrocytes depends on β-catenin while positive regulation is mediated by TCFs/LEF/ATF2 interaction. Cytokine.

[76] Brat D.J., Bellail A.C., Van Meir E.G. (2005). The role of interleukin-8 and its receptors in gliomagenesis and tumoral angiogenesis. Neuro-Oncol..

[77] Roebuck K.A. (1999). Regulation of interleukin-8 gene expression. J. Interferon Cytokine Res..

[78] Yu C., Narasipura S.D., Richards M.H., Hu X.T., Yamamoto B., Al-Harthi L. (2017). HIV and drug abuse mediate astrocyte senescence in a beta-catenin-dependent manner leading to neuronal toxicity. Aging Cell.

[79] Hou J., Kim S., Sung C., Choi C. (2017). Ginsenoside Rg3 prevents oxidative stress-induced astrocytic senescence and ameliorates senescence paracrine effects on glioblastoma. Molecules.

[80] Gao J., Liao Y., Qiu M., Shen W. (2020). Wnt/β-Catenin Signaling in Neural Stem Cell Homeostasis and Neurological Diseases. Neurosci. Rev. J. Bringing Neurobiol. Neurol. Psychiatry.

[81] Gonzalez H., Narasipura S.D., Shull T., Shetty A., Teppen T.L., Naqib A., Al-Harthi L. (2023). An Efficient and Cost-Effective Approach to Generate Functional Human Inducible Pluripotent Stem Cell-Derived Astrocytes. Cells.

[82] Chang C.-Y., Liang M.-Z., Wu C.-C., Huang P.-Y., Chen H.-I., Yet S.-F., Tsai J.-W., Kao C.-F., Chen L. (2020). WNT3A Promotes Neuronal Regeneration upon Traumatic Brain Injury. Int. J. Mol. Sci..

[83] Wei Z.Z., Zhang J.Y., Taylor T.M., Gu X., Zhao Y., Wei L. (2018). Neuroprotective and regenerative roles of intranasal Wnt-3a administration after focal ischemic stroke in mice. J. Cereb. Blood Flow Metab..

[84] Tassew N.G., Charish J., Shabanzadeh A.P., Luga V., Harada H., Farhani N., D’Onofrio P., Choi B., Ellabban A., Nickerson P.E.B. (2017). Exosomes Mediate Mobilization of Autocrine Wnt10b to Promote Axonal Regeneration in the Injured CNS. Cell Rep..

[85] Patel S., Alam A., Pant R., Chattopadhyay S. (2019). Wnt Signaling and Its Significance Within the Tumor Microenvironment: Novel Therapeutic Insights. Front. Immunol..

[86] Richards M.H., Narasipura S.D., Kim S., Seaton M.S., Lutgen V., Al-Harthi L. (2015). Dynamic interaction between astrocytes and infiltrating PBMCs in context of neuroAIDS. Glia.

[87] Wang Y., Su Y., Yu G., Wang X., Chen X., Yu B., Cheng Y., Li R., Sáez J.C., Yi C. (2021). Reduced Oligodendrocyte Precursor Cell Impairs Astrocytic Development in Early Life Stress. Adv. Sci..

[88] Yin K., Peluso M.J., Luo X., Thomas R., Shin M.-G., Neidleman J., Andrew A., Young K.C., Ma T., Hoh R. (2024). Long COVID manifests with T cell dysregulation, inflammation and an uncoordinated adaptive immune response to SARS-CoV-2. Nat. Immunol..

[89] Espín E., Yang C., Shannon C.P., Assadian S., He D., Tebbutt S.J. (2023). Cellular and molecular biomarkers of long COVID: A scoping review. eBioMedicine.

[90] Phetsouphanh C., Darley D.R., Wilson D.B., Howe A., Munier C.M.L., Patel S.K., Juno J.A., Burrell L.M., Kent S.J., Dore G.J. (2022). Immunological dysfunction persists for 8 months following initial mild-to-moderate SARS-CoV-2 infection. Nat. Immunol..

[91] Sorensen T.L., Trebst C., Kivisakk P., Klaege K.L., Majmudar A., Ravid R., Lassmann H., Olsen D.B., Strieter R.M., Ransohoff R.M. (2002). Multiple sclerosis: A study of CXCL10 and CXCR3 co-localization in the inflamed central nervous system. J. Neuroimmunol..

[92] Balashov K.E., Rottman J.B., Weiner H.L., Hancock W.W. (1999). CCR5^+^ and CXCR3^+^ T cells are increased in multiple sclerosis and their ligands MIP-1α and IP-10 are expressed in demyelinating brain lesions. Proc. Natl. Acad. Sci. USA.

[93] Franciotta D., Martino G., Zardini E., Furlan R., Bergamaschi R., Andreoni L., Cosi V. (2001). Serum and CSF levels of MCP-1 and IP-10 in multiple sclerosis patients with acute and stable disease and undergoing immunomodulatory therapies. J. Neuroimmunol..

[94] Bradburn S., McPhee J., Bagley L., Carroll M., Slevin M., Al-Shanti N., Barnouin Y., Hogrel J.Y., Pääsuke M., Gapeyeva H. (2018). Dysregulation of C-X-C motif ligand 10 during aging and association with cognitive performance. Neurobiol. Aging.

[95] Kutsch O., Oh J., Nath A., Benveniste E.N. (2000). Induction of the chemokines interleukin-8 and IP-10 by human immunodeficiency virus type 1 tat in astrocytes. J. Virol..

[96] Kolb S.A., Sporer B., Lahrtz F., Koedel U., Pfister H.-W., Fontana A. (1999). Identification of a T cell chemotactic factor in the cerebrospinal fluid of HIV-1-infected individuals as interferon-γ inducible protein 10. J. Neuroimmunol..

[97] Sanders V.J., Pittman C.A., White M.G., Wang G., Wiley C.A., Achim C.L. (1998). Chemokines and receptors in HIV encephalitis. Aids.

[98] Oh S.J., Kumari P., Auroni T.T., Stone S., Pathak H., Elsharkawy A., Natekar J.P., Shin O.S., Kumar M. (2024). Upregulation of Neuroinflammation-Associated Genes in the Brain of SARS-CoV-2-Infected Mice. Pathogens.

[99] Kumari P., Rothan H.A., Natekar J.P., Stone S., Pathak H., Strate P.G., Arora K., Brinton M.A., Kumar M. (2021). Neuroinvasion and Encephalitis Following Intranasal Inoculation of SARS-CoV-2 in K18-hACE2 Mice. Viruses.

[100] Carossino M., Kenney D., O’Connell A.K., Montanaro P., Tseng A.E., Gertje H.P., Grosz K.A., Ericsson M., Huber B.R., Kurnick S.A. (2022). Fatal Neurodissemination and SARS-CoV-2 Tropism in K18-hACE2 Mice Is Only Partially Dependent on hACE2 Expression. Viruses.

[101] Seehusen F., Clark J.J., Sharma P., Bentley E.G., Kirby A., Subramaniam K., Wunderlin-Giuliani S., Hughes G.L., Patterson E.I., Michael B.D. (2022). Neuroinvasion and Neurotropism by SARS-CoV-2 Variants in the K18-hACE2 Mouse. Viruses.

[102] Rothan H.A., Kumari P., Stone S., Natekar J.P., Arora K., Auroni T.T., Kumar M. (2022). SARS-CoV-2 Infects Primary Neurons from Human ACE2 Expressing Mice and Upregulates Genes Involved in the Inflammatory and Necroptotic Pathways. Pathogens.

