# Supplementary material for: SARS-CoV-2 Infection Influences Wnt/β-Catenin Pathway Components in Astrocytes
Source: Pathogens. 2025 Oct 2;14(10):994. doi: 10.3390/pathogens14100994 (PMC12567431; doi:10.3390/pathogens14100994)
Supplement: Supplementary file 1 [file pathogens-14-00994-s001.zip › pathogens-3822904-supplementary.pdf]

Supplementary Material:

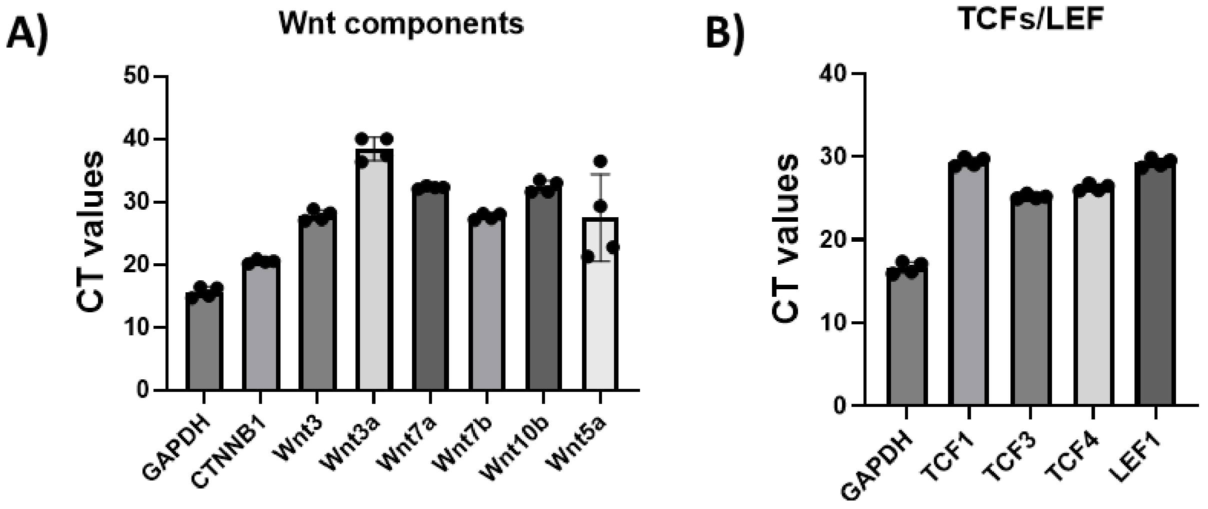

Supplementary Figure S1: Endogenous expression of Wnt signaling components in iAs. Endogenous expression of canonical and noncanonical Wnt ligands **(A)** and TCF/LEF **(B)** in iAs was measured by real-time PCR after 48h culture. Data is representative of 4 replicates. Error bars represent the SD.

A. CT values for mRNA in the Wnt pathway

	GAPDH	CTNNB1	Wnt3	Wnt3a	Wnt7a	Wnt7b	Wnt10b	Wnt5a
Number of values	4	4	4	4	4	4	4	4
Minimum	14.67	20.13	27.01	36.35	32.02	27.13	31.57	21.29
Maximum	16.46	20.89	28.85	40.00	32.58	28.13	33.48	36.46
Range	1.786	0.7597	1.840	3.651	0.5525	1.001	1.916	15.17
Mean	15.62	20.51	27.83	38.42	32.29	27.67	32.42	27.46
Std. Deviation	0.8984	0.3137	0.8592	1.865	0.2258	0.5067	0.9840	6.944
Std. Error of Mean	0.4492	0.1568	0.4296	0.9323	0.1129	0.2533	0.4920	3.472
Shapiro-Wilk test								
W	0.8561	0.9920	0.9215	0.8346	0.9559	0.8488	0.8327	0.9173
P value	0.2466	0.9674	0.5454	0.1800	0.7529	0.2223	0.1749	0.5217

Passed normality test (alpha=0.05)?	Yes	Yes	Yes	Yes	Yes	Yes	Yes	Yes
P value summary	ns	ns	ns	ns	ns	ns	ns	ns

B. CT values for mRNA in the TCF/LEF family

	GAPDH	TCF1	TCF3	TCF4	LEF1
Number of values	4	4	4	4	4
Minimum	15.90	28.85	24.94	25.89	28.68
Maximum	17.35	29.94	25.56	26.77	29.86
Range	1.453	1.088	0.6235	0.8824	1.181
Mean	16.62	29.39	25.18	26.27	29.26
Std. Deviation	0.7097	0.5314	0.2754	0.4299	0.5443
Std. Error of Mean	0.3549	0.2657	0.1377	0.2150	0.2722
Shapiro-Wilk test					
W	0.8864	0.8862	0.9092	0.8789	0.9406
P value	0.3665	0.3659	0.4782	0.3340	0.6578
Passed normality test (alpha=0.05)?	Yes	Yes	Yes	Yes	Yes
P value summary	ns	ns	ns	ns	ns

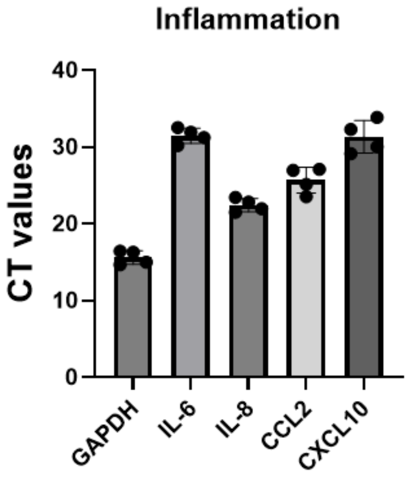

Supplementary Figure S2: Endogenous expression of inflammatory genes and type I interferons in iAs. Endogenous expression of inflammatory genes in iAs was measured by real-time PCR after 48h culture. Data is representative of 4 replicates. Error bars represent the SD.

Endogenous expression of inflammatory genes and type I interferons in iAs.

	GAPDH	IL-6	IL-8	CCL2	CXCL10
Number of values	4	4	4	4	4
Minimum	14.67	30.21	21.50	23.54	29.15
Maximum	16.46	32.54	23.46	27.11	33.87
Range	1.786	2.323	1.963	3.576	4.721
Mean	15.62	31.49	22.45	25.70	31.35
Std. Deviation	0.8984	1.002	0.8820	1.698	2.146
Std. Error of Mean	0.4492	0.5010	0.4410	0.8492	1.073
Shapiro-Wilk test					
W	0.8561	0.9792	0.9619	0.8830	0.9495
P value	0.2466	0.8972	0.7910	0.3515	0.7129
Passed normality test (alpha=0.05)?	Yes	Yes	Yes	Yes	Yes
P value summary	ns	ns	ns	ns	ns

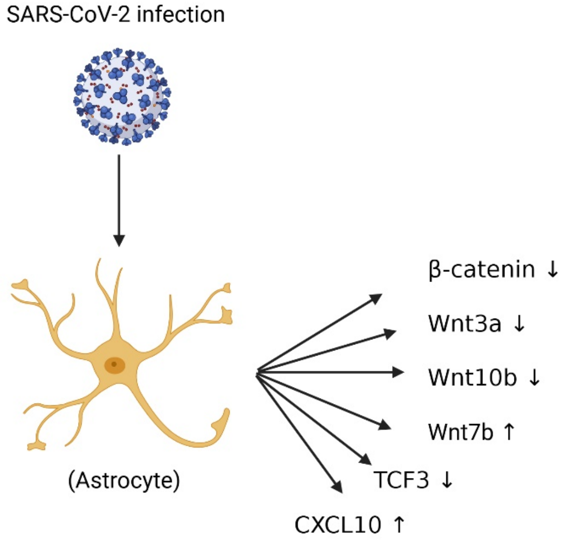

Supplementary Figure S3: SARS-CoV-2 direct infection of iAs in vitro alters multiple homeostatic and inflammatory factors. These changes are consistent with a complex cellular response to infection.

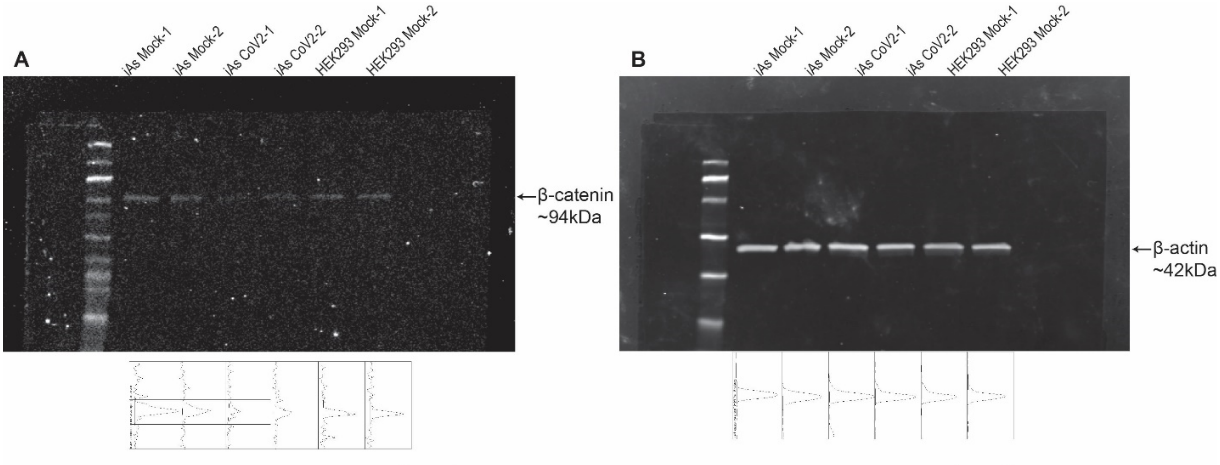

Supplementary Figure S4. Raw data and quantification for Western blots depicted in Figure 4. A) Western blot of the samples of iAs mock infected, iAs SARS-CoV-2 infected, and HEK293 mock infected, probed with anti-beta-Catenin antibody. HEK293 were included as a positive control for detection of beta-catenin. Below, density plots for each lane as generated by FIJI Gel Analyzer Macro. **B)** Western blot of the samples of iAs mock infected, iAs SARS-CoV-2 infected, and HEK293 mock infected, probed with anti-beta-actin antibody. Below, density plots for each lane.

Supplemental Material: Table of Statistical Analyses

Table S1: iAs cultures contain astrocytes as assessed by flow cytometry for GFAP.

Number of values	3
Minimum	85.10
Maximum	89.40
Range	4.300
Mean	87.87
Std. Deviation	2.401

Std. Error of Mean 1.386

Shapiro-Wilk test	
W	0.8021
P value	0.1194
Passed normality test (alpha=0.05)?	Yes
P value summary	ns

Table S2: SARS CoV2 Viral infection in astrocytes: genomes/ml
Kolmogorov-Smirnov test indicated experimental values have lognormal distribution.

Kolmogorov-Smirnov test		
KS distance	1.000	0.2334
P value	<0.0001	>0.1000
Passed lognormality test (alpha=0.05)?	No	Yes
P value summary	****	ns

We therefore used the one sample ratio t test to compare the geometric mean of our samples with the hypothetical geometric mean (i.e. the limit of detection).

One sample ratio t test	Sample difference has zero SD	
t, df		t=7.268, df=6
P value (two tailed)		0.0003
P value summary		***
Significant (alpha=0.05)?		Yes

Table S3: SARS-CoV-2 infectious units in astrocyte supernatant over time. For this experiment, data are plotted on log scale and analyzed by non-parametric analysis.

Kruskal-Wallis test	
P value	0.0025
Exact or approximate P value?	Approximate
P value summary	**
Do the medians vary signif. (P < 0.05)?	Yes
Number of groups	5
Kruskal-Wallis statistic	16.39

Number of families	1			
Number of comparisons per family	4			
Alpha	0.05			
Dunn's multiple comparisons test	Mean rank diff.	Significant?	Summary	Adjusted P Value
Unt. vs. SARS-CoV-2 24h	-13.50	Yes	**	0.0060
Unt. vs. SARS-CoV-2 48h	-12.75	Yes	*	0.0108
Unt. vs. SARS-CoV-2 72h	-11.00	Yes	*	0.0386
Unt. vs. SARS-CoV-2 96h	-10.75	Yes	*	0.0457

Test details	Mean rank 1	Mean rank 2	Mean rank diff.	n1
Unt. vs. SARS-CoV-2 24h	4.500	18.00	-13.50	8
Unt. vs. SARS-CoV-2 48h	4.500	17.25	-12.75	8
Unt. vs. SARS-CoV-2 72h	4.500	15.50	-11.00	8
Unt. vs. SARS-CoV-2 96h	4.500	15.25	-10.75	8

Table S4. Ctnnb1 mRNA fold change.

Kolmogorov-Smirnov test demonstrated normal distribution of data.

Kolmogorov-Smirnov test		
KS distance	0.1701	0.2268
P value	>0.1000	>0.1000
Passed normality test (alpha=0.05)?	Yes	Yes
P value summary	ns	ns

Unpaired t-test demonstrated significant effect of infection.

Unpaired t test	
P value	0.0002
P value summary	***
Significantly different (P < 0.05)?	Yes
One- or two-tailed P value?	Two-tailed
t, df	t=6.505, df=8

Table S5. Wnt3 mRNA.

Kolmogorov-Smirnov test		
KS distance	0.1148	0.2092
P value	>0.1000	>0.1000
Passed normality test (alpha=0.05)?	Yes	Yes
P value summary	ns	ns
Number of values	5	5

Unpaired t test	
P value	0.1510
P value summary	ns
Significantly different (P < 0.05)?	No
One- or two-tailed P value?	Two-tailed
t, df	t=1.588, df=8

Table S6. Fold change in Wnt3a mRNA.

Kolmogorov-Smirnov test		
KS distance	0.3255	0.2331
P value	0.0898	>0.1000
Passed normality test (alpha=0.05)?	Yes	Yes
P value summary	ns	ns

Unpaired t test	
P value	0.0166

P value summary	*
Significantly different (P < 0.05)?	Yes
One- or two-tailed P value?	Two-tailed
t, df	t=3.020, df=8
How big is the difference?	
Mean of column A	0.8985
Mean of column B	0.2986
Difference between means (B - A) ± SEM	-0.5999 ± 0.1987
95% confidence interval	-1.058 to -0.1418
R squared (eta squared)	0.5326
F test to compare variances	
F, DFn, Dfd	1.927, 4, 4
P value	0.5409
P value summary	ns
Significantly different (P < 0.05)?	No

Table S7. Fold change in Wnt7a mRNA.

Kolmogorov-Smirnov test		
KS distance	0.3376	0.2544
P value	0.0641	>0.1000
Passed normality test (alpha=0.05)?	Yes	Yes
P value summary	ns	ns

Variances were unequal so Welch's correction was applied to the unpaired t test.

Unpaired t test with Welch's correction	
P value	0.3967
P value summary	ns
Significantly different (P < 0.05)?	No
One- or two-tailed P value?	Two-tailed
Welch-corrected t, df	t=0.9384, df=4.399
How large is the effect?	
Mean of column A	0.9746
Mean of column B	1.397
Difference between means (B - A) ± SEM	0.4228 ± 0.4506
95% confidence interval	-0.7847 to 1.630
R squared (eta squared)	0.1668
F test to compare variances	
F, DFn, Dfd	19.98, 4, 4
P value	0.0132
P value summary	*
Significantly different (P < 0.05)?	Yes

Table S8. Fold change in Wn7b mRNA.

Kolmogorov-Smirnov test		
KS distance	0.1927	0.1404
P value	>0.1000	>0.1000
Passed normality test (alpha=0.05)?	Yes	Yes

P value summary	ns	ns
Unpaired t test		
P value	<0.0001	
P value summary	****	
Significantly different (P < 0.05)?	Yes	
One- or two-tailed P value?	Two-tailed	
t, df	t=7.518, df=10	
How big is the difference?		
Mean of column A	1.010	
Mean of column B	1.738	
Difference between means (B - A) ± SEM	0.7276 ± 0.09677	
95% confidence interval	0.5119 to 0.9432	
R squared (eta squared)	0.8497	
F test to compare variances		
F, DFn, Dfd	1.209, 5, 5	
P value	0.8400	
P value summary	ns	
Significantly different (P < 0.05)?	No	

Table S9. Fold change in Wnt10b mRNA.

Kolmogorov-Smirnov test		
KS distance	0.2485	0.2637
P value	>0.1000	>0.1000
Passed normality test (alpha=0.05)?	Yes	Yes
P value summary	ns	ns
Unpaired t test		
P value	0.0049	
P value summary	**	
Significantly different (P < 0.05)?	Yes	
One- or two-tailed P value?	Two-tailed	
t, df	t=3.840, df=8	
How large is the effect?		
Mean of column A	1.185	
Mean of column B	0.3374	
Difference between means (B - A) ± SEM	-0.8479 ± 0.2208	
95% confidence interval	-1.357 to -0.3387	
R squared (eta squared)	0.6482	
F test to compare variances		
F, DFn, Dfd	2.035, 4, 4	
P value	0.5084	
P value summary	ns	
Significantly different (P < 0.05)?	No	

Table S10: Fold change in beta-Catenin protein as analyzed by Western blot.

Column B	SARS-CoV-2
vs.	vs.

Column A		Mock infected
Unpaired t test		
P value		0.0279
P value summary		*
Significantly different (P < 0.05)?		Yes
One- or two-tailed P value?		Two-tailed
t, df		t=2.884, df=6
How large is the effect?		
Mean of column A		0.7778
Mean of column B		0.4113
Difference between means (B - A) ± SEM		-0.3665 ± 0.1271
95% confidence interval		-0.6774 to -0.05556
R squared (eta squared)		0.5810
F test to compare variances		
F, DFn, Dfd		4.358, 3, 3
P value		0.2579
P value summary		ns
Significantly different (P < 0.05)?		No
Shapiro-Wilk test for Normality		
W	0.9882	0.9246
P value	0.9483	0.5631
Passed normality test (alpha=0.05)?	Yes	Yes
P value summary	ns	ns

Table S11: Cells positive for beta-Catenin protein as analyzed by intracellular staining flow cytometry.

Shapiro-Wilk test for normal distribution			
W	0.8456	0.9995	
P value	0.2286	0.9593	
Passed normality test (alpha=0.05)?	Yes	Yes	
P value summary	ns	ns	
Unpaired t test with Welch's correction			
P value			0.1958
P value summary			ns
Significantly different (P < 0.05)?			No
One- or two-tailed P value?			Two-tailed
Welch-corrected t, df			t=1.887, df=2.066
How big is the difference?			
Mean of column A			78.60
Mean of column B			42.90
Difference between means (B - A) ± SEM			-35.70 ± 18.92
95% confidence interval			-114.7 to 43.27
R squared (eta squared)			0.6327
F test to compare variances			
F, DFn, Dfd			60.28, 2, 2
P value			0.0326
P value summary			*
Significantly different (P < 0.05)?			Yes

Table S12. Fold change in TCF1 mRNA.

Kolmogorov-Smirnov test			
KS distance	0.1419	0.2084	
P value	>0.1000	>0.1000	
Passed normality test (alpha=0.05)?	Yes	Yes	
P value summary	ns	ns	
Unpaired t test			
P value			0.7859
P value summary			ns
Significantly different (P < 0.05)?			No
One- or two-tailed P value?			Two-tailed
t, df			t=0.2809, df=8
How big is the difference?			
Mean of column A			1.015
Mean of column B			0.9628
Difference between means (B - A) ± SEM			-0.05180 ± 0.1844
95% confidence interval			-0.4771 to 0.3735
R squared (eta squared)			0.009765
F test to compare variances			
F, DFn, Dfd			3.563, 4, 4
P value			0.2461
P value summary			ns
Significantly different (P < 0.05)?			No

Table S13. Fold change in TCF3 mRNA.

Kolmogorov-Smirnov test		
KS distance	0.3616	0.3145
P value	0.0314	>0.1000
Passed normality test (alpha=0.05)?	No	Yes
P value summary	*	ns
Mann Whitney test		
P value	0.0238	
Exact or approximate P value?	Exact	
P value summary	*	
Significantly different (P < 0.05)?	Yes	
One- or two-tailed P value?	Two-tailed	
Sum of ranks in column A,B	38.50 , 16.50	
Mann-Whitney U	1.500	
Difference between medians		
Median of column A	1.000, n=5	
Median of column B	0.7007, n=5	
Difference: Actual	-0.2993	
Difference: Hodges-Lehmann	-0.3370	

Table S14. Fold change in TCF4 mRNA.

Kolmogorov-Smirnov test		
KS distance	0.3034	0.1915
P value	>0.1000	>0.1000
Passed normality test (alpha=0.05)?	Yes	Yes
P value summary	ns	ns
Unpaired t test		
P value	0.5831	
P value summary	ns	
Significantly different (P < 0.05)?	No	
One- or two-tailed P value?	Two-tailed	
t, df	t=0.5718, df=8	
How big is the difference?		
Mean of column A	1.012	
Mean of column B	0.9013	
Difference between means (B - A) ± SEM	-0.1109 ± 0.1939	
95% confidence interval	-0.5581 to 0.3363	
R squared (eta squared)	0.03927	
F test to compare variances		
F, DFn, Dfd	5.033, 4, 4	
P value	0.1466	
P value summary	ns	
Significantly different (P < 0.05)?	No	

Table S15. Fold change in LEF1 mRNA.

Kolmogorov-Smirnov test		
KS distance	0.1606	0.2692
P value	>0.1000	>0.1000
Passed normality test (alpha=0.05)?	Yes	Yes
P value summary	ns	ns
Unpaired t test with Welch's correction		
P value	0.5205	
P value summary	ns	
Significantly different (P < 0.05)?	No	
One- or two-tailed P value?	Two-tailed	
Welch-corrected t, df	t=0.6809, df=6.203	
How big is the difference?		
Mean of column A	1.004	
Mean of column B	1.209	
Difference between means (B - A) ± SEM	0.2041 ± 0.2998	
95% confidence interval	-0.5236 to 0.9319	
R squared (eta squared)	0.06954	
F test to compare variances		
F, DFn, Dfd	59.03, 6, 6	

P value	<0.0001	
P value summary	****	
Significantly different (P < 0.05)?	Yes	

Table S16. IL6 mRNA.

Kolmogorov-Smirnov test		
KS distance	0.1895	0.2369
P value	>0.1000	>0.1000
Passed normality test (alpha=0.05)?	Yes	Yes
P value summary	ns	ns
Unpaired t test with Welch's correction		
P value	0.5454	
P value summary	ns	
Significantly different (P < 0.05)?	No	
One- or two-tailed P value?	Two-tailed	
Welch-corrected t, df	t=0.6566, df=4.243	
How large is the effect?		
Mean of column A	1.024	
Mean of column B	1.447	
Difference between means (B - A) ± SEM	0.4238 ± 0.6454	
95% confidence interval	-1.328 to 2.176	
R squared (eta squared)	0.09223	
F test to compare variances		
F, DFn, Dfd	32.87, 4, 4	
P value	0.0051	
P value summary	**	
Significantly different (P < 0.05)?	Yes	

Table S17. IL8 mRNA

Kolmogorov-Smirnov test		
KS distance	0.1783	0.2118
P value	>0.1000	>0.1000
Passed normality test (alpha=0.05)?	Yes	Yes
P value summary	ns	ns
Unpaired t test		
P value	0.4228	
P value summary	ns	
Significantly different (P < 0.05)?	No	
One- or two-tailed P value?	Two-tailed	
t, df	t=0.8448, df=8	
How big is the difference?		
Mean of column A	1.007	

Mean of column B	1.071	
Difference between means (B - A) ± SEM	0.06463 ± 0.07651	
95% confidence interval	-0.1118 to 0.2411	
R squared (eta squared)	0.08191	
F test to compare variances		
F, DFn, Dfd	1.351, 4, 4	
P value	0.7777	
P value summary	ns	
Significantly different (P < 0.05)?	No	

Table S18. Fold change in CCL2 mRNA.

Kolmogorov-Smirnov test		
KS distance	0.1260	0.1901
P value	>0.1000	>0.1000
Passed normality test (alpha=0.05)?	Yes	Yes
P value summary	ns	ns
Unpaired t test with Welch's correction		
P value	0.2653	
P value summary	ns	
Significantly different (P < 0.05)?	No	
One- or two-tailed P value?	Two-tailed	
Welch-corrected t, df	t=1.270, df=4.532	
How large is the effect?		
Mean of column A	1.003	
Mean of column B	1.198	
Difference between means (B - A) ± SEM	0.1949 ± 0.1534	
95% confidence interval	-0.2120 to 0.6017	
R squared (eta squared)	0.2626	
F test to compare variances		
F, DFn, Dfd	14.98, 4, 4	
P value	0.0225	
P value summary	*	
Significantly different (P < 0.05)?	Yes	

Table S19. Fold change in CXCL10 mRNA.

Kolmogorov-Smirnov test		
KS distance	0.1774	0.2147
P value	>0.1000	>0.1000
Passed normality test (alpha=0.05)?	Yes	Yes
P value summary	ns	ns
Unpaired t test with Welch's correction		
P value	0.0014	

P value summary	**
Significantly different (P < 0.05)?	Yes
One- or two-tailed P value?	Two-tailed
Welch-corrected t, df	t=7.103, df=4.463
How big is the difference?	
Mean of column A	1.016
Mean of column B	3.775
Difference between means (B - A) ± SEM	2.759 ± 0.3884
95% confidence interval	1.723 to 3.795
R squared (eta squared)	0.9187
F test to compare variances	
F, DFn, Dfd	17.20, 4, 4
P value	0.0174
P value summary	*
Significantly different (P < 0.05)?	Yes

Table S20. CXCL10 ELISA.

Kolmogorov-Smirnov test			
KS distance	0.1196	0.1586	
P value	>0.1000	>0.1000	
Passed normality test (alpha=0.05)?	Yes	Yes	
P value summary	ns	ns	
Unpaired t test with Welch's correction			
P value			0.0307
P value summary			*
Significantly different (P < 0.05)?			Yes
One- or two-tailed P value?			Two-tailed
Welch-corrected t, df			t=2.474, df=11.14
How large is the effect?			
Mean of column A			12.77
Mean of column B			16.63
Difference between means (B - A) ± SEM			3.860 ± 1.561
95% confidence interval			0.4309 to 7.290
R squared (eta squared)			0.3546
F test to compare variances			
F, DFn, Dfd			4.897, 8, 8
P value			0.0375
P value summary			*
Significantly different (P < 0.05)?			Yes

Table S21. Cytotoxicity in infected astrocytes by assay for LDH secretion.

Test for normal distribution

Shapiro-Wilk test		
W	0.9460	0.9602
P value	0.5521	0.6165
Passed normality test (alpha=0.05)?	Yes	Yes
P value summary	ns	ns
Number of values	3	3

Unpaired t test	
P value	0.7950
P value summary	ns
Significantly different (P < 0.05)?	No
One- or two-tailed P value?	Two-tailed
t, df	t=0.2777, df=4
How large is the effect?	
Mean of column B	0.4730
Mean of column C	0.4853
Difference between means (C - B) ± SEM	0.01233 ± 0.04442
95% confidence interval	-0.1110 to 0.1357
R squared (eta squared)	0.01891
F test to compare variances	
F, DFn, Dfd	5.658, 2, 2
P value	0.3004
P value summary	ns
Significantly different (P < 0.05)?	No

Table S22. Cell death in infected astrocytes by flow cytometry staining with Zombie violet.

Shapiro-Wilk test		
W	0.9067	0.9313
P value	0.4072	0.4933
Passed normality test (alpha=0.05)?	Yes	Yes
P value summary	ns	ns

Unpaired t test	
P value	0.0776
P value summary	ns
Significantly different (P < 0.05)?	No
One- or two-tailed P value?	Two-tailed
t, df	t=2.361, df=4
How large is the effect?	
Mean of column A	82.73
Mean of column B	75.50
Difference between means (B - A) ± SEM	-7.233 ± 3.063

95% confidence interval	-15.74 to 1.272
R squared (eta squared)	0.5823
